# TGF-β Affects the Differentiation of Human GM-CSF^+^ CD4^+^ T Cells in an Activation- and Sodium-Dependent Manner

**DOI:** 10.3389/fimmu.2016.00603

**Published:** 2016-12-23

**Authors:** Szabolcs Éliás, Angelika Schmidt, Venkateshan Kannan, John Andersson, Jesper Tegnér

**Affiliations:** ^1^Unit of Computational Medicine, Center for Molecular Medicine, Department of Medicine Solna, Karolinska University Hospital and Science for Life Laboratory, Karolinska Institutet, Stockholm, Sweden; ^2^Immunology and Allergy Unit, Department of Medicine Solna, Karolinska Institutet, Stockholm, Sweden

**Keywords:** human CD4^+^ T cells, differentiation, GM-CSF, TGF-β, autoimmune diseases, multiple sclerosis, multivariate analysis, sodium

## Abstract

The cytokine granulocyte-macrophage colony-stimulating factor (GM-CSF) is involved in the pathogenesis of chronic inflammatory diseases such as multiple sclerosis. However, the environmental cues promoting differentiation of GM-CSF producing T cells are unclear. Herein, we performed a broad experimental screening of cytokines and data-driven analysis assessing their ability to induce human GM-CSF^+^ CD4^+^ T cells and their subpopulations. TGF-β was discovered to induce GM-CSF production independently of proliferation and IL-2 signaling including STAT5. In contrast, IL-6 and IL-23 decreased GM-CSF production. On the population level, GM-CSF induction was highly correlated with expression of FOXP3 across cytokine stimulations but not with that of IL-17. However, on single-cell level GM-CSF and IFN-γ expression were most correlated, independently of the cytokine environment. Importantly, under low sodium conditions in the medium or upon stimulation with plate-bound instead of bead-bound anti-CD3 and anti-CD28 antibodies, the effects of TGF-β on GM-CSF, but not on FOXP3, were reversed. Our analysis indicates a novel role for TGF-β in generating GM-CSF^+^ subsets of human CD4^+^ T cells. These results are important for understanding of autoimmune disease and therapeutic considerations.

## Introduction

CD4^+^ T cells are central in directing immune responses and have been implicated in numerous diseases such as chronic inflammation, cancer, and neurodegenerative diseases. CD4^+^ T cells can be classified as effector T helper (T_h_) cells and immunosuppressive regulatory T cells (T_reg_). T_h_ cells can be further divided into the major subsets T_h1_, T_h2_, and T_h17_ cells, based on their cytokine profile such as expression of IFN-γ, IL-4, and IL-17 that is driven largely by expression of the lineage-defining transcription factors T-bet, GATA-3, and RORγt, respectively (1). The most important T_reg_ subset, comprised of thymus-derived and peripherally induced T_regs_, is characterized by expression of the lineage-defining transcription factor FOXP3 (2). However, recent investigations revealed numerous subsets as well as plasticity in the CD4^+^ T cell population beyond the classical distinction between T_h1_, T_h2_, T_h17_, and T_reg_ (3, 4). For example, T_h9_, T_h22_, T_fh_, T_fr_ have been described as additional subpopulations (1).

Another emerging T_h_ cell subset is defined by the production of granulocyte-macrophage colony-stimulating factor (GM-CSF). So far no lineage-defining transcription factor has been identified for this subpopulation. In CD4^+^ T cells GM-CSF can be co-expressed with other lineage-defining cytokines such as IFN-γ, but GM-CSF single-positive cells have also been identified (5). The importance of GM-CSF in inflammatory disorders is illustrated by the fact that GM-CSF^−/−^ mice are completely resistant to experimental autoimmune encephalomyelitis (EAE), an animal model for multiple sclerosis (MS) (6, 7). GM-CSF produced by auto-reactive T cells is necessary for the onset of EAE (8) unlike IFN-γ and IL-17 (7, 8). In human cerebrospinal fluid, the fraction of GM-CSF^+^ and IFN-γ^+^ cells within CD4^+^ T cells was elevated in MS patients compared to controls whereas the fraction of IL-17^+^ cells was not significantly different (5). In peripheral blood, the fraction of GM-CSF^+^ and IFN-γ^+^ cells within CD4^+^ T cells was elevated in MS patients compared to controls in one study (9) but not in another (5). Interestingly, myelin-reactive T cells – present in MS patients and healthy donors with comparable frequencies – have been determined by single-cell cloning to produce more IFN-γ, IL-17, and GM-CSF and less IL-10 in MS patients compared to healthy controls (10).

It has been proposed from cell transfer experiments in EAE that T_h17_ cells have “non-pathogenic T_h17_” and “pathogenic T_h17_” subclasses and that the latter expresses GM-CSF (11–13). However, the factors inducing pathogenic CD4^+^ T cells and the markers defining them on the single-cell level are incompletely understood. IL-1β, IL-23, TGF-β3, and NaCl have been proposed as induction factors of murine pathogenic T_h17_ cells (11–16). Notably IL-23 – a cytokine not produced by T cells – has a well-known role in inducing autoimmune disease, a fact that has been linked to its effect on T_h17_ cells. Under T_h17_-polarizing conditions, IL-1β and IL-23 induce GM-CSF^+^IL-17^+^ double-positive murine cells (14). T_h17_ cells generated under addition of TGF-β3 or elevated NaCl concentrations displayed enhanced expression of *Csf2* (the gene encoding for GM-CSF) on the population level but have not been studied at single-cell resolution (11, 13). Another study on the contrary found that neither addition of TGF-β1 nor TGF-β3 rendered murine T_h17_ cells pathogenic, possibly due to insufficient GM-CSF production (17).

Together, the identity of pathogenic CD4^+^ T cells remains obscure, while the importance of T cell-produced GM-CSF is undisputed. Pathogenicity cannot be tested in humans and it appears that there are differences in human compared to murine GM-CSF^+^ T cells. For example, on the level of single CD4^+^ T cells, IL-17 and GM-CSF can be co-expressed in murine cells (14), whereas their expression was mutually exclusive in human cells (5). Regarding factors inducing GM-CSF in human CD4^+^ T cells, TGF-β1 or TGF-β3 was found to decrease GM-CSF production in one study (9), while TGF-β1 had no effect in another (5). IL-23 and IL-6 did not augment GM-CSF (5, 9), whereas IL-2 or IL-7 signaling induced GM-CSF expression in a STAT5-dependent manner and IL-1β induced IFN-γ^+^ GM-CSF^+^ double-positive cells (5, 9).

Together, the results of the above studies support a role of GM-CSF^+^ CD4^+^ T cells in MS but despite their importance in disease, the differentiation factors and characteristics of human GM-CSF^+^ CD4^+^ T cells are poorly defined and seem to be different from the ones in mouse. Here, we screened several cytokines in various combinations for their ability to induce GM-CSF^+^ cells from human naïve CD4^+^ T cells. We found that TGF-β was the most potent inducer of GM-CSF^+^ CD4^+^ T cells, which was also dependent on the mode of T cell activation and independent of IL-2 signaling. In contrast, IL-23 and IL-6 inhibited GM-CSF production. GM-CSF^+^ cells comprised several subpopulations and were induced under similar conditions as FOXP3^+^ cells on the population level while on single-cell level, IFN-γ was most strongly correlated with GM-CSF. Notably, under low sodium conditions, the effects of TGF-β on GM-CSF induction were reversed. Our results shed light on the cytokine, medium, and stimulation conditions required to induce human GM-CSF^+^ T cells and their phenotype regarding subpopulations, which may contribute to the understanding of their role in human autoimmune disease in the future.

## Materials and Methods

### Cell Isolation

Human peripheral blood mononuclear cells (PBMCs) were isolated using Ficoll-Paque gradient centrifugation. In brief, buffy coats diluted in PBS were overlaid on Ficoll-Paque and centrifuged at 1200 × *g* for 20 min without break and the PBMC ring was collected. Cells were washed with PBS (450 × *g*, 10 min) and monocytes depleted by adherence to plastic culture flasks. Human naïve CD4^+^ T cells were isolated using the human naive CD4^+^ T cell isolation kit (Miltenyi) according to the manufacturer’s instructions. The purity of naïve CD4^+^ T cells was controlled by flow cytometry and was above 94%.

### *In Vitro* T Cell Differentiation

Human naïve CD4^+^ T cells were cultured in 96-well round bottom plates in serum-free X-VIVO 15 medium (Lonza) with a final sodium concentration of 145.8 mM (by addition of 30 mM NaCl) and activated using Dynabeads Human T-Activator anti-CD3-, anti-CD28-coated beads (Invitrogen) at bead:cell ratio of 1:1 in the presence of the specified cytokines and 10 µg/ml each anti-IFN-γ (RnD systems) and anti-IL-4 (RnD systems) blocking antibodies for 5 days unless otherwise stated. The sodium concentration in blood plasma is (135 to) 145 mM Na^+^. Addition of 30 mM NaCl to X-VIVO 15 medium resembles this physiological Na^+^ concentration (here termed “physiologic” sodium conditions) and X-VIVO 15 medium supplemented in this way has been used by others to culture CD4^+^ T cells (18, 19). In some experiments (termed “low sodium” conditions), no additional NaCl was added to the X-VIVO 15 medium (which contains 115.8 mM total sodium). In some experiments, cells were activated with 5 µg/ml plate-bound (pb) anti-CD3 (clone OKT3; Biolegend, LEAF grade) and 1 µg/ml soluble anti-CD28 antibody (clone CD28.2; Biolegend, LEAF grade). Cytokines (all from RnD Systems) were used at the following concentrations unless otherwise stated: IL-1β (12.5 ng/ml), IL-6 (25 ng/ml), IL-21 (25 ng/ml, Life technologies), IL-23 (25 ng/ml), IL-2 (100 IU/ml), IL-10 (5 or 25 ng/ml), TGF-β1 (5 ng/ml), and TGF-β3 (5 ng/ml). Where indicated, STAT5 inhibitor (*N*′-((4-Oxo-4H-chromen-3-yl)methylene)nicotinohydrazide, CAS No.: 285986-31-4, Merck Millipore/Calbiochem) at 200 µM or anti-IL-2-blocking antibody (BD Pharmingen, clone: MQ1-17H12, no azide/low endotoxin) at 5 µg/ml or respective isotype control antibody was added.

### Flow Cytometry

Naïve CD4^+^ T cell purity was verified by surface staining using anti-CD45RA-FITC (Miltenyi, Clone: T6D11) and anti-CD4-APC (eBioscience, Clone: OKT4) antibodies followed by flow cytometry.

Viability staining and proliferation staining were done using Fixable Viability Dye eFluor 780 (eBioscience) and Cell Proliferation Dye eFluor 450 (eBioscience), respectively, according to the manufacturer’s instructions.

Prior to all intracellular stainings, cells were restimulated for 5 h using 10 ng/ml phorbol 12-myristate 13-acetate (PMA) and 375 ng/ml ionomycin (Iono) in the presence of 1× GolgiPlug containing Brefeldin A (BFA; BD Biosciences) to block protein secretion. Then, cells were resuspended thoroughly and magnetic anti-CD3/anti-CD28-coated beads were removed on a magnet. Intracellular stainings were performed using the FOXP3 Transcription Factor Staining Buffer Set (eBioscience) according to the manufacturer’s instructions.

The following antibodies (all against the human proteins) were used: GM-CSF-APC (Miltenyi, clone: BVD2-21C11), GM-CSF-PE-CF594 (BD Biosciences, clone BVD2-21C11), FOXP3-FITC (eBioscience, clone: 236A/E7), FOXP3-eFluor450 (eBioscience, clone: 236A/E7), FOXP3-APC (eBioscience, clone: 236A/E7), IFN-γ-FITC (eBioscience, clone: 4S.B3), IFN-γ-PE-Vio770 (eBioscience, clone: 45-15), IL-17A-PE (eBioscience, clone: Bio64DEC17), CD25-PE (Miltenyi, clone: 4E3), IL-2-PE-CF594 (BD Biosciences, clone: 5344.111), CD4-PE-CF594 (BD Biosciences, clone: RPA-T4). The following antibodies were used for isotype control staining: Mouse IgG1κ Isotype Control-FITC (eBioscience, clone: P3.6.2.8.1), Mouse IgG1κ Isotype Control-PE (eBioscience, clone: P3.6.2.8.1), Mouse IgG1κ Isotype Control-PE-Vio770 (Miltenyi, clone: IS5-21F5), Mouse IgG1κ Isotype Control-PE-CF594 (BD Biosciences, clone: X40), Mouse IgG1κ Isotype Control-APC (eBioscience, clone: P3.6.2.8.1), Rat IgG2aκ Isotype Control-PE-CF594 (BD Biosciences, clone: R35-95), Rat IgG2aκ Isotype Control-APC (eBioscience, clone: eBR2a).

Flow Cytometry recording was performed on a CyAn ADP 9-Color Analyzer (Beckman Coulter), and compensation settings were created automatically with the CyAn software (Summit) using single stained compensation beads (BD CompBeads, BD Biosciences) containing both positive and negative populations.

Flow cytometry data analysis was performed using the R programming language for statistical computing including the flowCore (20), flowStats (21) R packages, or with the FlowJo software (Tree Star). Gating strategy involved the following sequential steps: gating on lymphocytes based on forward and side scatter, doublet-exclusion based on pulse width *versus* forward scatter area, gating on live cells (Fixable Viability Dye-eFluor780 negative population), and in case when CD4 staining was used gating on CD4^+^ population.

### Enzyme-Linked Immunosorbent Assay (ELISA)

Sandwich ELISA for human GM-CSF was performed with the Human GM-CSF Mini ABTS ELISA Development Kit (Peprotech) along with ABTS substrate (Sigma-Aldrich) according to the manufacturer’s instructions.

### Statistical Analysis

The R programming language was used for statistical analysis. When data were not normally distributed according to Shapiro–Wilk test, the non-parametric Wilcoxon signed-rank test was used paired within each donor to determine statistical significance for comparing groups. Significance is presented as *p*-value.

### Model Selection Using LASSO Regression for Regularization

Linear regression with shrinkage (least absolute shrinkage and selection operator, LASSO) was carried out using the glmnet (22) R package. The target variable was the difference in the GM-CSF^+^ cell fraction within live cells between the cytokine-treated sample and the control (activation without cytokines) for every given donor. The full set of predictors (prior selection) were defined as (i) cytokines that were applied in the stimulation experiments, (ii) interactions between all pairs of cytokines in the stimulation experiments, and (iii) time. The predictors were standardized and transformed in the following way: the concentration values of each cytokine (i) and time (iii) were all normalized between values 0 and 1 in order to prevent detection of differential effects between predictors simply due to the fact that they are in different ranges. Normalization was done by linear scaling of the range of the values as follows:

$$f(x)=\frac{x-\text{min}(x)}{\text{max}(x)-\text{min}(x)}$$

The interaction values between cytokine pairs (ii) were calculated in the following way: first calculating the product of the corresponding normalized cytokine values (i). Since the product of two uniform distributions ranging between 0 and 1 is a non-uniform distribution, the second step was to apply a transformation that converts the non-uniform product distribution into a uniform distribution. The following equation was used for this transformation:

f(x)=x−xln(x)

This was derived in the following way: the distribution of the product of *n* uniformly distributed random variables on [0,1] is given by $P(x)=\frac{-1^{n-1}}{\left(n-1\right)!}\ln{\left(x\right)}^{n-1}$. The desired transformation function is then precisely the integral of this probability density. Carrying out the integral for *n* = 2 gives the above transformation function.

Ten-fold cross-validation (CV) with squared error loss was used to determine the shrinkage parameter λ. However, different realizations of the fold-split led to different CV errors and hence, mean CV error was obtained from 1,000 runs of CV to identify the optimal λ.

Predicted net effects are given as (i) the coefficient values of the regression model for individual cytokines and (ii) the sum of the individual cytokine coefficients and their interaction coefficient for cytokine pairs.

### t-SNE and Model-Based Clustering

For t-SNE and clustering analysis, equivalent numbers of data points from three donors per condition were pooled. These samples were recorded using the same voltage settings on the flow cytometer. Fluorescence intensity values of each marker were normalized by linear scaling as described above for LASSO regression. t-SNE for dimensionality reduction (23) was performed using the Rtsne (24) R package and the following settings: maximum number of iterations = 10,000, perplexity = 258 (that is 10% of the total number of data points), theta = 0 (exact t-SNE).

Model-based clustering was performed using the flowClust (25, 26) R package with the following settings: Gaussian mixture models were used (ν = ∞), convergence tolerance of expectation-maximization (EM) = 10^−15^, maximum number of EM iterations = 10 000, points outside 80% quantile region were considered as outliers (out of cluster). The number of clusters was chosen so that (i) the value of Bayesian information criterion was close to zero and at the same time (ii) the number of clusters was minimal.

### Ethics Statement

Peripheral blood mononuclear cells were freshly isolated from anonymized healthy donor buffy coats purchased from the Karolinska University Hospital (Karolinska Universitetssjukhuset, Huddinge), Sweden. Research was performed according to the national Swedish ethical regulations (ethical review act, SFS number 2003:460).

## Results

### Induction of GM-CSF^+^ CD4^+^ T Cells Is Promoted by TGF-β and Inhibited by IL-6 and IL-23

To investigate conditions for induction of GM-CSF^+^ cells, we cultured human naïve CD4^+^ T cells in the presence of (i) IL-6, IL-21, and TGF-β1 that have been shown to be involved in the induction of IL-17^+^ cells (27–29), (ii) IL-1β, IL-23, and TGF-β3 that have been proposed to induce murine “pathogenic” GM-CSF^+^IL-17^+^ cells (11, 14–16), and (iii) IL-2 that has been reported to induce human GM-CSF^+^ CD4^+^ T cells (5, 9). Along with these cytokines, the cells were activated with anti-CD3-, anti-CD28-coated beads (anti-CD3/CD28 beads) for 5–6 days, and GM-CSF expression was measured by intracellular flow cytometry. We cultured the cells in serum-free X-VIVO 15 medium with, if not otherwise stated, a final sodium concentration of 145.8 mM, which we consider “physiologic” sodium levels similar to the concentration in blood (135–145 mM). As demonstrated by others before, addition of 10–40 mM NaCl to X-VIVO 15 medium is suitable for differentiation cultures of human CD4^+^ T cells and is equivalent to the concentrations found in the interstitium of mice on high-salt diet (18, 19, 30). Importantly, these increased NaCl concentrations were required to detect substantial fractions of T_h17_ cells, and the authors demonstrated that osmolarity was not causing this effect but specifically sodium was responsible for inducing “pathogenic” T_h17_ cells (13, 18). We chose to add 30 mM NaCl (145.8 mM final sodium) in our experiments to represent conditions allowing for T_h17_ induction and at the same time maintaining high cell viability (as measured by flow cytometry; data not shown) and physiological sodium concentrations.

**Figure 1:**
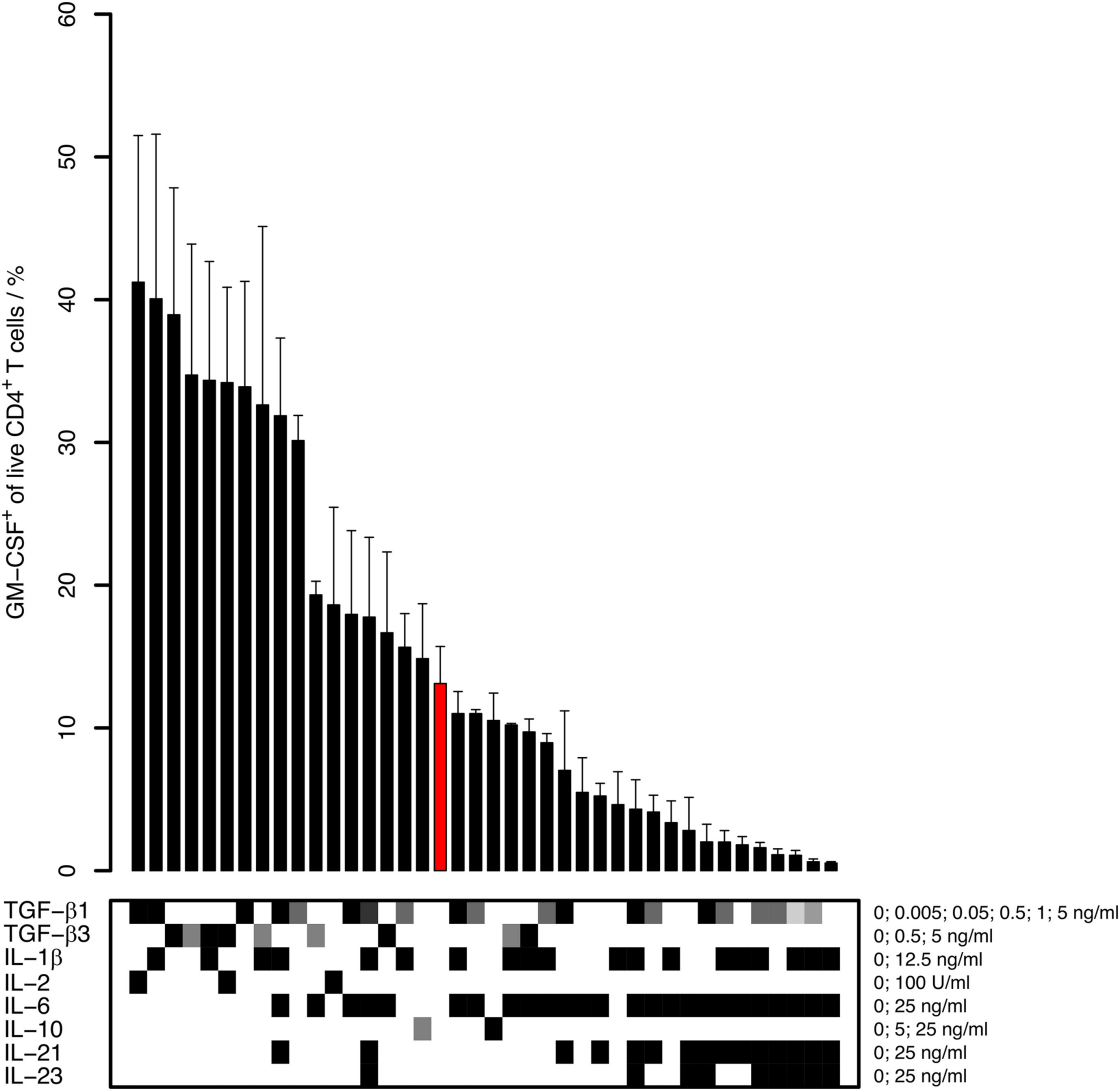
**Capability of different cytokines to induce human GM-CSF^+^ CD4^+^ T cells**. Human naïve CD4^+^ T cells were activated with anti-CD3/CD28 beads in the presence of the indicated cytokines for 5–6 days. The percentage of GM-CSF^+^ cells within live CD4^+^ T cells was measured by intracellular flow cytometry after 5 h of restimulation with phorbol 12-myristate 13-acetate (PMA) and Ionomycin in the presence of Brefeldin A (PMA/Iono/BFA). Bars represent mean + SEM of 2–15 donors. The lower panel represents cytokine concentrations (white: absence of cytokine; gray to black: low to high concentrations with the concentrations and units given on the right side of the table). The bar highlighted in red represents the reference, that is, stimulation with anti-CD3/CD28 in medium without cytokine addition (No cytokine).

**Figure 2:**
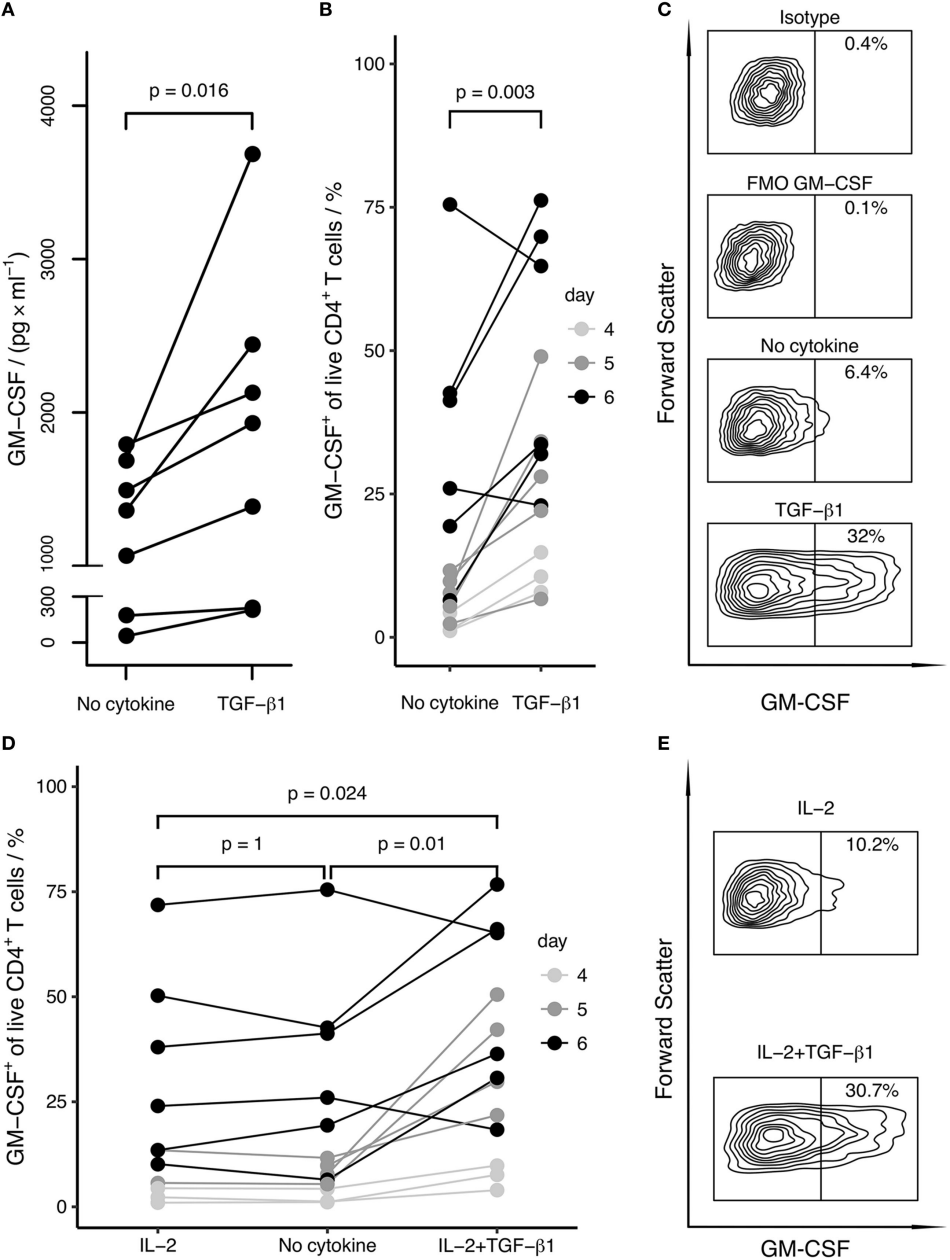
**TGF-β enhances and IL-2 does not affect the differentiation of GM-CSF^+^ CD4^+^ T cells**. Human naïve CD4^+^ T cells were activated with anti-CD3/CD28 beads in the presence of the indicated cytokines for 4–6 days. GM-CSF expression was either measured in the supernatant or cells were restimulated with PMA/Iono/BFA, and the percentage of GM-CSF^+^ cells within live CD4^+^ T cells was measured by intracellular flow cytometry. **(A)** After stimulation for 5 days, levels of secreted GM-CSF in the cell culture supernatant from cells activated in the absence (No cytokine) or presence of TGF-β1 (TGF-β1) were measured by ELISA. Replicate well cell platings were measured separately, ELISA readings for replicate wells were averaged and dots show the mean values. Each line represents a single donor. **(B)** The percentage of GM-CSF^+^ cells among live CD4^+^ T cells is shown comparing activation (No cytokine) and activation in the presence of TGF-β1 (TGF-β1) for the indicated time periods. Each line represents a single donor. **(C)** As an example for a donor from **(B)**, flow cytometry contour plots of the following samples gated on live cells are shown from one donor after 6 days of stimulation: isotype control for GM-CSF (Isotype), fluorescence-minus-one control for GM-CSF (FMO GM-CSF), activation alone (No cytokine), activation in the presence of TGF-β1 (TGF-β1). **(D)** The percentage of GM-CSF^+^ cells among live CD4^+^ T cells is shown comparing the effects of activation in the presence of IL-2 (IL-2), activation alone (No cytokine), and activation in the presence of IL-2 and TGF-β1 (IL-2 + TGF-β1). Each line represents a single donor; symbols as in **(B)**. **(E)** As an example for **(D)**, flow cytometry contour plots of the following samples gated on live cells are shown from one donor after 6 days of stimulation: activation in the presence of IL-2 (IL-2), activation in the presence of IL-2 and TGF-β1 (IL-2 + TGF-β1). Statistical significance was calculated using non-parametric paired Wilcoxon signed-rank test (*p*-values are indicated).

Under these conditions, we found that the induction of GM-CSF^+^ cells was enhanced by TGF-β and was inhibited by pro-inflammatory cytokines, in particular by IL-6 and IL-23 (Figure 1). The anti-inflammatory cytokine IL-10 did neither markedly increase nor decrease GM-CSF expression. TGF-β1 and TGF-β3 had similar effects on GM-CSF induction, and we therefore further focused our investigations on TGF-β1 because this is the most abundant isoform throughout the human body (31). TGF-β1 increased the secretion of GM-CSF in cell culture supernatants, as measured by ELISA (Figure 2A). In unstimulated naïve CD4^+^ T cells, GM-CSF was detectable neither in the supernatant nor in PMA/Iono/BFA-pulsed or un-pulsed cells in flow cytometry (data not shown). When assaying GM-CSF expression at different time points of activation, we confirmed that TGF-β1 significantly enhanced the fraction of GM-CSF^+^ cells (Figures 2B,C). Taken together, these data demonstrate that TGF-β can increase GM-CSF expression in differentiating human CD4^+^ T cells.

### The GM-CSF-Inducing Effect of TGF-β Is Independent of IL-2 Signaling and Proliferation

**Figure 3:**
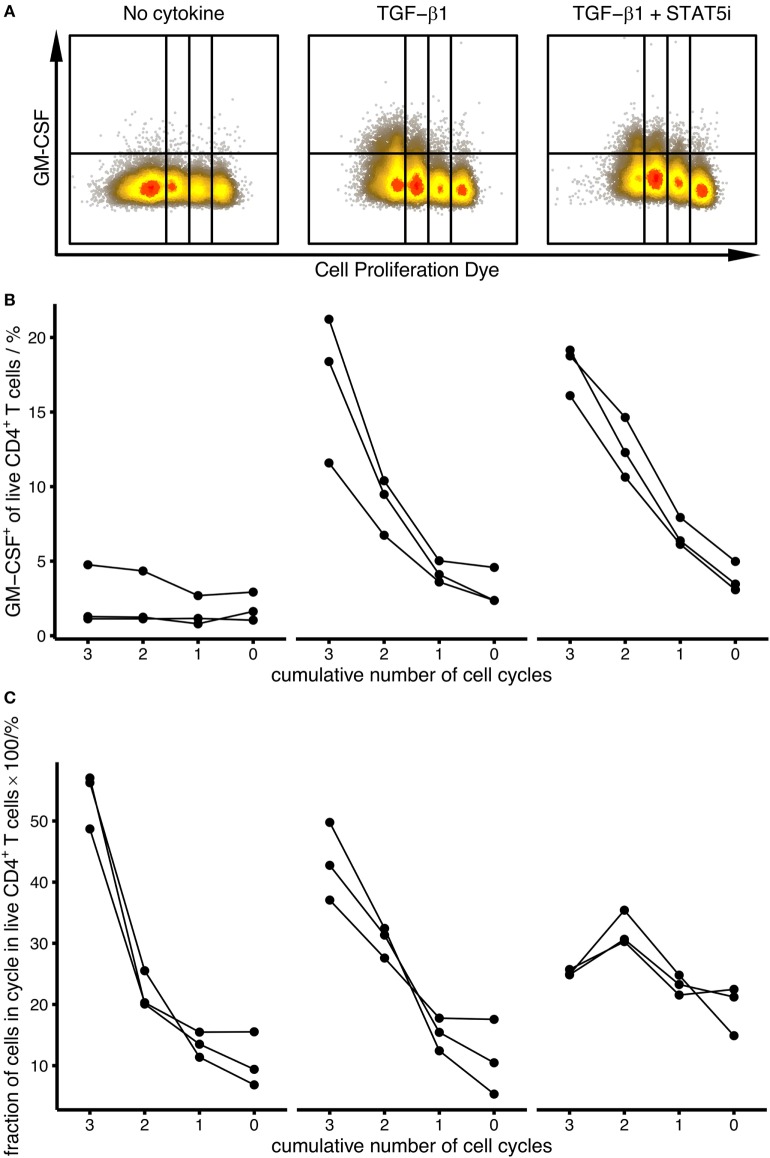
**Induction of human GM-CSF^+^ CD4^+^ T cells by TGF-β is independent of STAT5 and proliferation**. Human naïve CD4^+^ T cells were labeled using Cell Proliferation Dye eFluor450 and were activated with anti-CD3/CD28 beads alone (No cytokine), in the presence of TGF-β1 (TGF-β1) or in the presence of TGF-β1 plus STAT5 inhibitor (TGF-β1 + STAT5i) for 4 days. The percentage of GM-CSF^+^ cells within live CD4^+^ T cells within each cycle was measured by intracellular flow cytometry after restimulation with PMA/Iono/BFA. **(A)** Representative dot plots for each stimulation condition. **(B)** Percentage of GM-CSF^+^ cells within the given proliferation cycles is shown for each stimulation condition [as labeled in **(A)**]. Each line represents a single donor. **(C)** Distribution of cell populations across proliferation cycles is shown for each stimulation condition as in **(A)**. Each line represents a single donor.

IL-2 is abundantly produced by activated CD4^+^ T cells, and it has been proposed that IL-2 is capable to induce GM-CSF expression in differentiating human CD4^+^ T cells (5, 9). Therefore, we considered the possibility that TGF-β may enhance IL-2 expression or IL-2 responsiveness, which in turn might induce the expression of GM-CSF. To inspect this possibility, we either added IL-2 to the culture or neutralized secreted IL-2 with a blocking antibody. Addition of IL-2 did not increase GM-CSF^+^ cell fractions (Figures 2D,E). Importantly, TGF-β1 had an enhancing effect on GM-CSF cell fractions also in the presence of IL-2 (Figures 2D,E). Furthermore, neutralization of IL-2 in the supernatant had no effect on TGF-β1-induced GM-CSF expression; however, this method of IL-2 blocking did not affect proliferation despite use of previously published (5) or higher concentrations of blocking antibody (Figure S1 in Supplementary Material and data not shown). Furthermore, we used a STAT5 inhibitor and observed that STAT5 inhibition did not prevent GM-CSF induction by TGF-β, corroborating that TGF-β-induced expression of GM-CSF is independent of IL-2 signaling (Figures 3A,B). At the same time as expected, STAT5 inhibition reduced proliferation since proliferation is enhanced by IL-2 signaling (Figure 3C).

Since we observed that GM-CSF expression was higher in proliferating cells, we investigated whether TGF-β1 increased the percentage of GM-CSF^+^ cells merely by increasing their proliferation. Therefore, we compared the percentage of GM-CSF^+^ cells within each proliferation cycle thereby conditioning on proliferation and eliminating its possibly confounding effects. Compared to activation without TGF-β1, TGF-β1 induced higher fractions of GM-CSF^+^ cells in every proliferation cycle (and in the presence of STAT5 inhibitor), suggesting that this effect is independent of proliferation (Figures 3A,B). Together, our data show that GM-CSF expression is enhanced by TGF-β independently of the proliferation state and IL-2 signaling.

### TGF-β-Induced GM-CSF Expression Is Dependent on the Mode of T Cell Activation

**Figure 4:**
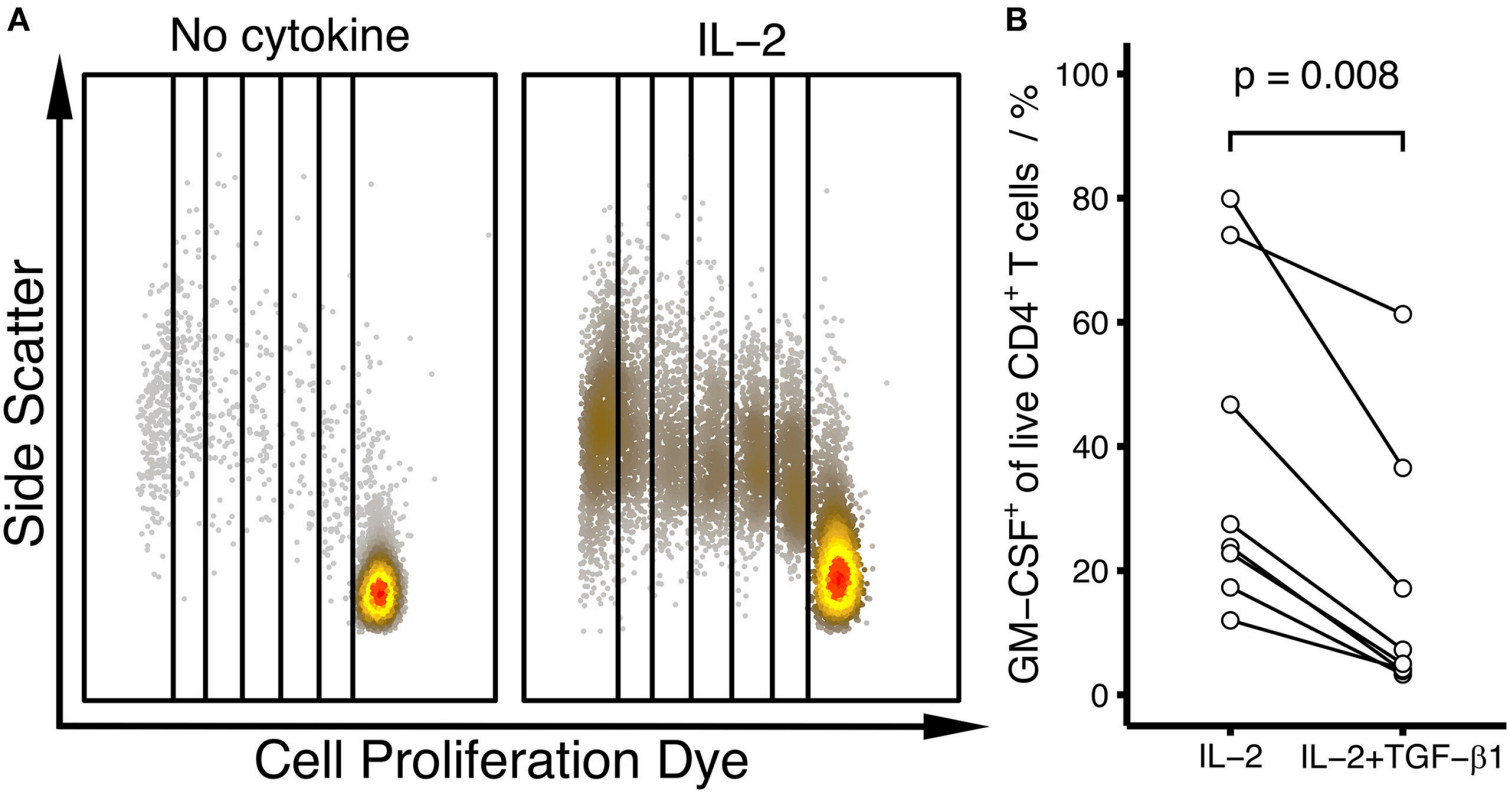
**The effect of TGF-β on the differentiation of human GM-CSF^+^ CD4^+^ T cells is dependent on the type of activation**. Human naïve CD4^+^ T cells were labeled using Cell Proliferation Dye eFluor450 and were activated with pb anti-CD3 and soluble anti-CD28 antibodies alone (No cytokine), in the presence of IL-2 (IL-2) or in the presence of IL-2 plus TGF-β1 (IL-2 + TGF-β1) for 6 days. **(A)** Dot plots showing proliferation in the absence (No cytokine) or presence of IL-2 (IL-2). Data from an example donor are shown. **(B)** The percentage of GM-CSF^+^ cells within live, blasted CD4^+^ T cells was measured by intracellular flow cytometry after restimulation with PMA/Iono/BFA, comparing the effects of IL-2 alone (IL-2) and IL-2 plus TGF-β1 (IL-2 + TGF-β1). Each line represents a single donor. Statistical significance was calculated using non-parametric paired Wilcoxon signed-rank test (*p*-values are indicated).

Because TGF-β has been shown to have no or inhibitory effects on GM-CSF expression in other studies (5, 9), we asked whether the activation conditions could affect the TGF-β-mediated effect on GM-CSF. In our experiments showing augmentation of GM-CSF expression by TGF-β, we used stimulation with bead-bound anti-CD3/CD28 antibodies, which induced robust proliferation of T cells (Figures 3). When we used pb anti-CD3 plus soluble anti-CD28 antibodies instead, this stimulation failed to induce robust proliferation of the cells but instead proliferation required external addition of IL-2 (Figure 4A). Under these conditions of pb anti-CD3 and soluble anti-CD28 stimulation plus IL-2, we observed that TGF-β reduced GM-CSF^+^ cell fractions (Figure 4B), suggesting that the GM-CSF-enhancing effect of TGF-β is dependent on the mode of T cell activation.

### TGF-β-Induced GM-CSF Expression Is Dependent on the Sodium Concentration

**Figure 5:**
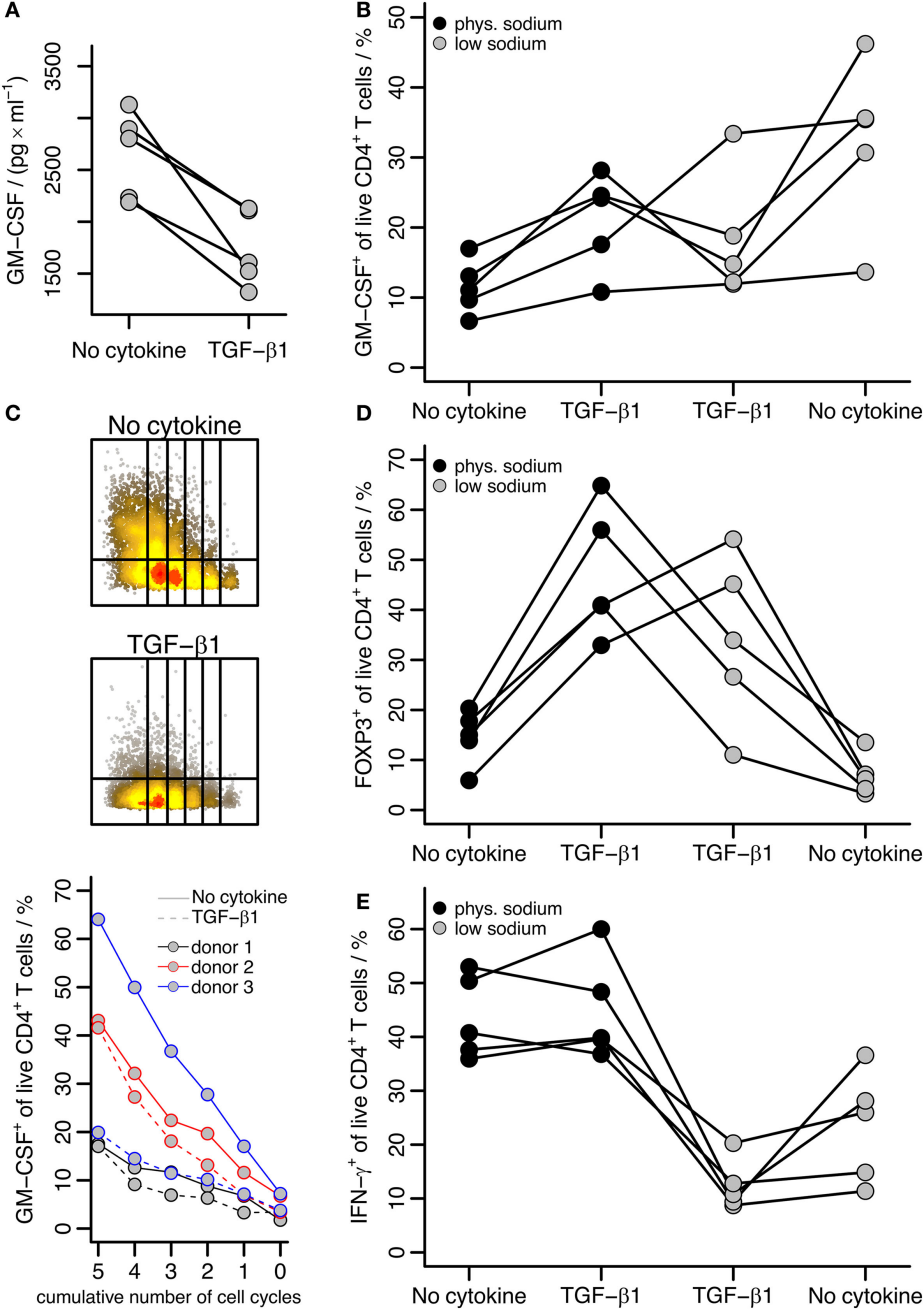
**The effect of TGF-β on GM-CSF induction is affected by the sodium concentration**. Human naïve CD4^+^ T cells were activated with anti-CD3/CD28 beads in the presence or absence of TGF-β1 for 5 days. A fraction of the supernatant was removed for ELISA, and then cells were restimulated with PMA/Iono/BFA and GM-CSF, IFN-γ, and FOXP3 within live CD4^+^ T cells were measured. **(A)** Levels of secreted GM-CSF were measured by ELISA for cells cultured under low sodium conditions; data are displayed according to the description in Figure 2A. **(B)** The percentage of GM-CSF^+^ cells among live CD4^+^ T cells is shown comparing activation (No cytokine) and activation in the presence of TGF-β1 (TGF-β1) either under “physiologic” (phys.) or “low” sodium concentrations as indicated. Each line represents a single donor. **(C)** Human naïve CD4^+^ T cells (labeled with Cell Proliferation Dye eFluor450) were activated with anti-CD3/CD28 beads alone (No cytokine) or in the presence of TGF-β1 (TGF-β1) under low sodium conditions for 5 days. The percentage of GM-CSF^+^ cells within live CD4^+^ T cells within each cycle was measured by intracellular flow cytometry after restimulation with PMA/Iono/BFA. The upper panels show flow cytometry raw data from one donor for both stimulation conditions, the lower panel shows the percentage of GM-CSF^+^ cells within the given proliferation cycles with lines colored according to donor and TGF-β1 addition indicated by dashed lines. **(D)** FOXP3^+^ and **(E)** IFN-γ^+^ cell fractions among live CD4^+^ T cells were determined and are presented as in **(B)**.

As described above, we cultured T cells under “physiologic” sodium conditions (145.8 mM total sodium) representing slight hypernatremia compared to blood but levels reasonable in interstitium. To compare with “low sodium” conditions, we cultured cells as above with anti-CD3/CD28 beads with or without TGF-β1 but without addition of sodium chloride, that is at 115.8 mM sodium final concentration. Interestingly, the TGF-β effect that induced GM-CSF under “physiologic” sodium concentrations was reversed in these “low sodium” experiments: under low sodium conditions, TGF-β reduced GM-CSF expression regarding the total amount of secreted GM-CSF (Figure 5A) as well as regarding the fraction of GM-CSF^+^ cells (Figure 5B), which was the reverse effect of TGF-β1 as compared to the effect under “physiologic” sodium levels (Figure 2) even when directly compared within the same donors (Figure 5B). In the absence of TGF-β, GM-CSF^+^ cell fractions were increased in low sodium compared to physiologic sodium concentrations (Figure 5B). The inhibitory effect of TGF-β1 on GM-CSF under low sodium conditions was independent of proliferation, as it was observed throughout multiple proliferation cycles (Figure 5C). Notably, we observed that TGF-β increased FOXP3 [a well-known effect (31)] irrespective of NaCl addition to the medium (Figure 5D). Correspondingly, a slight inhibition of IFN-γ by TGF-β, which is a known effect (32–34), seemed to occur under low sodium conditions; however in the presence of physiological sodium concentrations, the fractions of IFN-γ^+^ cells were generally much higher and an effect of TGF-β was not apparent (Figure 5E). Together, these results suggest that the effect of TGF-β specifically on GM-CSF expression is affected by sodium concentrations in the medium.

### GM-CSF Has Similar Induction Requirements as FOXP3 on the Population Level

**Figure 6:**
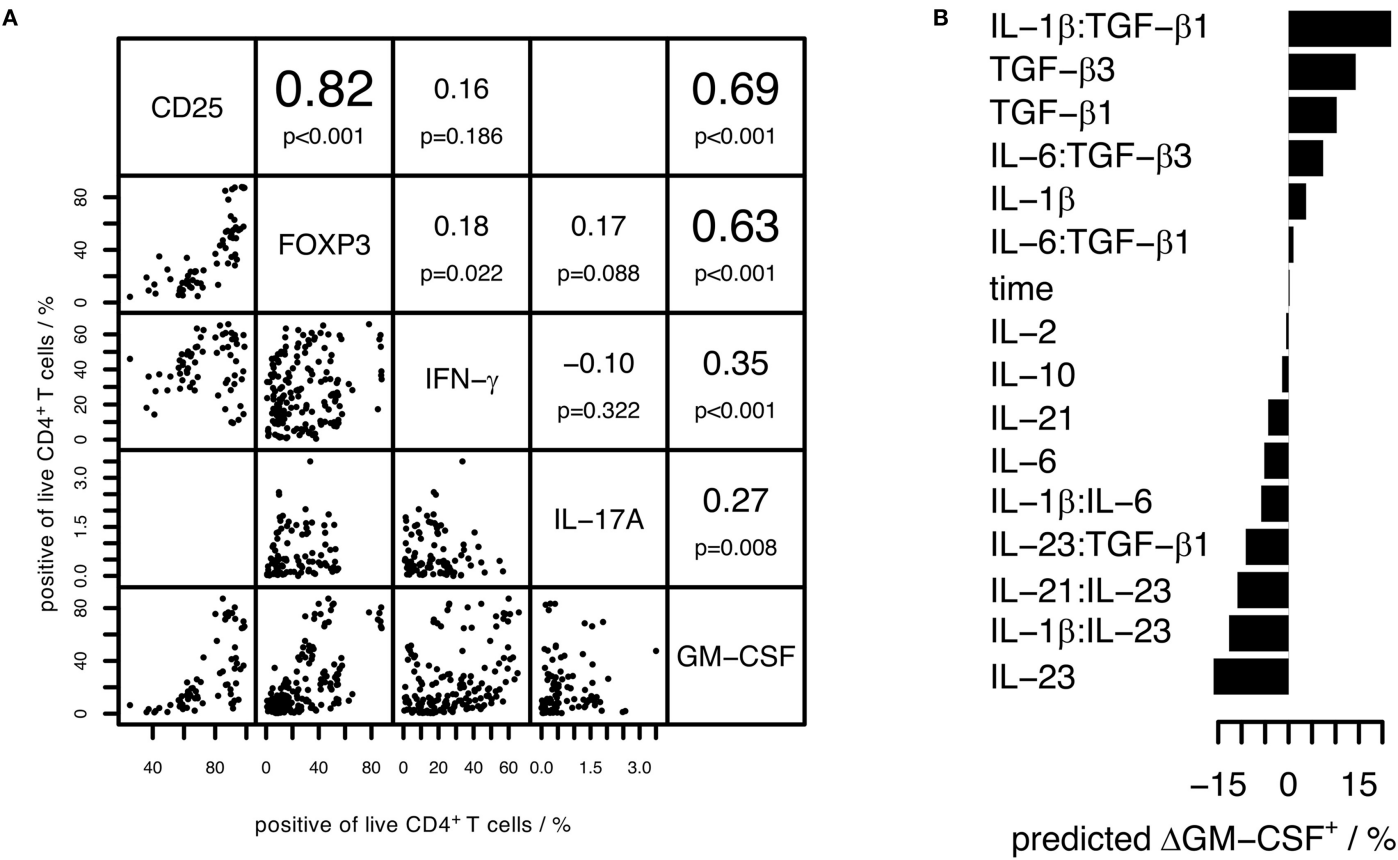

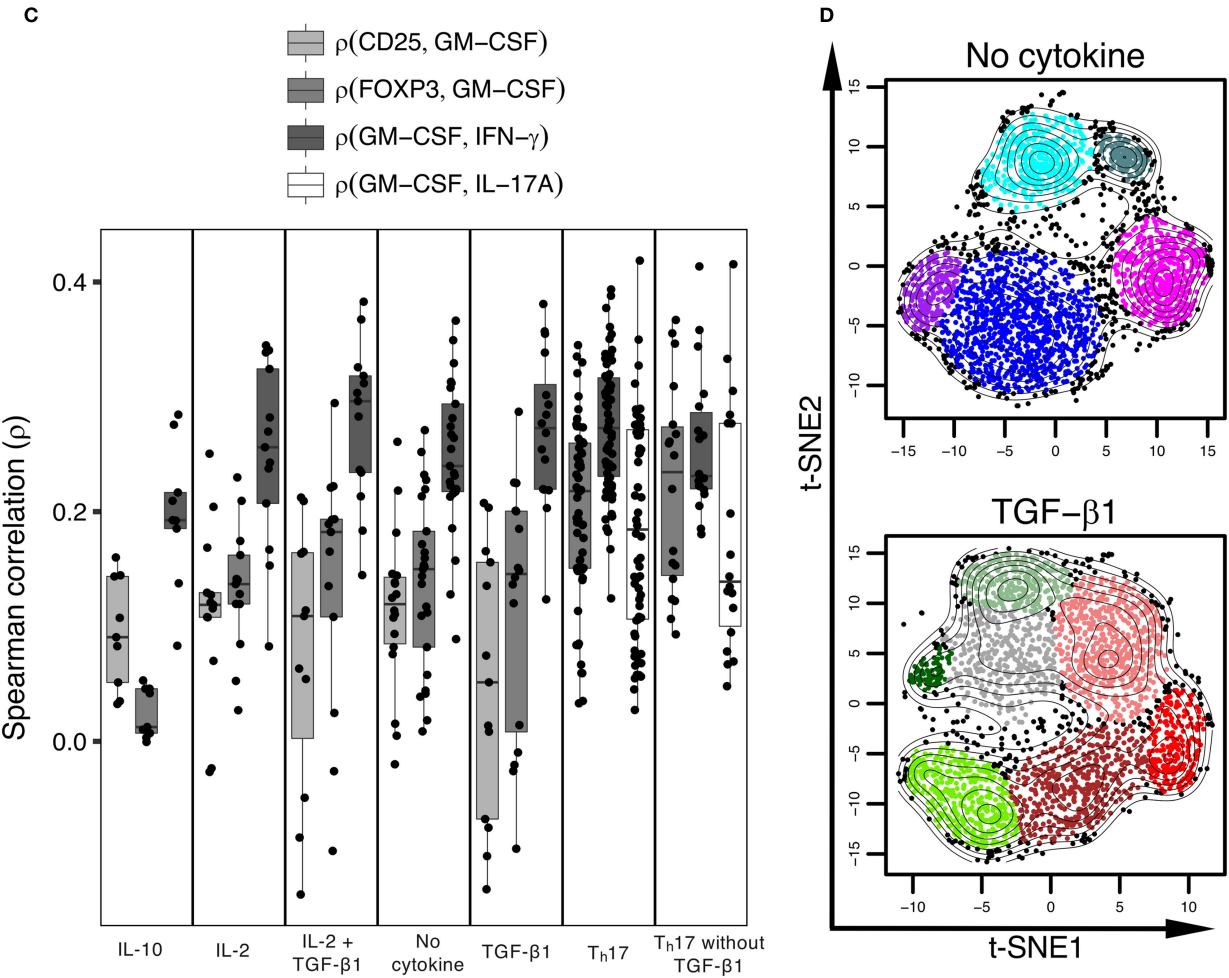
**The induction conditions of GM-CSF^+^ and FOXP3^+^ CD4^+^ T cells are similar, but on the single-cell level GM-CSF and IFN-γ are most co-expressed**. Human naïve CD4^+^ T cells were activated with anti-CD3/CD28 beads in the presence of the cytokines indicated in Figure 1 for 3–7 days. GM-CSF, IL-17A, IFN-γ, FOXP3, and CD25 within live CD4^+^ T cells were measured. **(A)** Correlation analysis based on the percentage positive cells for each marker across different stimulation conditions. Diagonal: markers considered in the analysis. Left to the diagonal: pairwise dot plots of markers showing percentage of positive cells across samples. Each sample is represented by a single dot and is defined by stimulation condition, donor, and experiment. Right to the diagonal: upper value denotes the Spearman correlation coefficient of percentage positive cells for the given pairs of markers, lower value denotes the *p*-value calculated using a paired *t*-test considering the Spearman correlation coefficient (ρ) as random variable and testing the null-hypothesis that it is zero (*H*_0_: ρ = 0). **(B)** The change in the GM-CSF^+^ cell fraction within live cells compared to the control (No cytokine) was considered as target variable and the normalized cytokine concentrations (including interaction effects) and time (3–7 days of activation) were considered as predictors in LASSO linear regression. Predicted net effects of cytokines and cytokine pairs on the change in the GM-CSF^+^ cell fraction compared to control are shown for the selected model. **(C)** Correlation analysis using normalized single-cell fluorescence intensity values of each marker. Samples were grouped based on stimulation condition, and the Spearman correlation between the indicated pairs of markers was calculated on the single-cell level and is shown as boxplot [samples as in **(A)**]. **(A–C)** Data from 2 to 15 donors per group were used. **(D)** Cells were stimulated with TGF-β1 (TGF-β1) or without (No cytokine). Pre-gated on live GM-CSF^+^ cells, t-SNE analysis of subpopulations (as specified in Figure S4 in Supplementary Material) followed by model-based clustering was performed. Data from three donors per group was pooled.

In previous studies, a fraction of GM-CSF^+^ cells has been found to co-express some other cytokines, but this has not been tested on single-cell level in all studies (5, 11, 13, 14, 18, 35). Single-positive GM-CSF^+^ cells have been suggested to be a separate subset not expressing other T cell subset markers (5). The co-expression of GM-CSF with other T cell markers has not been analyzed previously in an unbiased manner with computational tools. Therefore, to characterize which T cell subset markers are co-regulated with GM-CSF, we analyzed the correlations between pairs of GM-CSF^+^ cell fractions and cell fractions positive for CD25, IFN-γ, IL-17A, or FOXP3, respectively, under physiologic sodium concentrations and across several cytokine stimulation conditions (Figure 6A, cytokine conditions as in Figure 1). Across conditions, the CD25^+^ and FOXP3^+^ fractions were most strongly correlated with the GM-CSF^+^ fractions, followed by the IFN-γ^+^ and IL-17A^+^ fractions. This shows that on the population level, among the given set of “T cell subset” markers, FOXP3 is most co-regulated with GM-CSF throughout the cytokine stimulations applied. This suggests that GM-CSF and FOXP3 are induced by similar cytokines and the well-known FOXP3-inducing effect of TGF-β (31) corresponds to our observation that TGF-β induces GM-CSF.

To rigorously identify the induction and repression requirements for GM-CSF, we applied LASSO regularized regression on our data where we considered the change in percentage of GM-CSF^+^ cells (compared to control cells activated without cytokine) as target variable and the normalized concentration of added cytokines as predictors (see Materials and Methods and Figure 1). Details of the model selection are provided in Figure S2 in Supplementary Material. From this analysis, we discovered that TGF-β was primarily responsible for the induction of GM-CSF, and IL-6 and IL-23 for its repression (Figure 6B), thus confirming the patterns observed in Figure 1. We carried out the same analysis for all the given markers, and for FOXP3 the inducing and repressing factors were very similar to those of GM-CSF, when we considered the change in the percentage of FOXP3^+^ cells as target variable (data not shown).

### GM-CSF Is Most Strongly Co-Expressed with IFN-γ on the Single-Cell Level

The above analyses examined the correlation of GM-CSF with other markers at bulk cell population level across the inducing conditions, but that does not reveal to what extent any two markers are expressed by the same cell. Information about co-expression of other markers with GM-CSF on the single-cell level is however important considering the crucial role of GM-CSF^+^ T cells in EAE induction (6–8) and the unclear role of “pathogenic” T_h17_ cells, particularly in the human system. High correlation between positive cell fractions defined based on a pair of markers across stimulation conditions does not necessarily imply that these markers are expressed by the very same cells. To find the marker most preferentially co-expressed by the same cells as GM-CSF, we calculated the Spearman correlations between pairs composed of GM-CSF and either FOXP3, CD25, IFN-γ, or IL-17A, across single cells, under the conditions given in Figure 1. To visualize our results and to account for potentially different results based on certain types of inducing cytokines, we divided the conditions into subsets (defined in Figure 6C). We determined that across single cells it was IFN-γ that was most correlated with GM-CSF irrespective of the inducing cytokine condition, while FOXP3 was less correlated (Figure 6C). Importantly, even under T_h17_-inducing conditions, either with or without TGF-β, IL-17A had the lowest correlation with GM-CSF on single-cell level (Figure 6C). Together, these results demonstrate that among the set of markers that we investigated, GM-CSF is most preferentially produced by the same single cells as IFN-γ throughout a range of different cytokine conditions.

To confirm these results in a completely independent study and at the same time on mRNA level in murine *in vivo* generated cells, we analyzed publicly available single-cell RNA-sequencing data from T_h17_ cells published by Regev and colleagues (36). Here, IL-17A^+^ cells were sorted (based on GFP reporter expression) from mice at the peak of EAE disease from the central nervous system (CNS) and lymph nodes (LN), respectively. Using the data from these single sorted T_h17_ cells, we analyzed on the single-cell level the correlation of expression of the gene encoding for GM-CSF (*Csf2*) with the genes encoding for the markers (IFN-γ, FOXP3, IL-17, CD25) that we studied in human cells before. Notably, despite being sorted based on IL-17A expression, the single cells displayed a range of *Il17a* and *Il17f* expression including non-expressing cells, which allowed us to analyze the correlation of *Csf2* also with *Il17a* and *Il17f* (Figures S3A,B in Supplementary Material). *Csf2* expression in CNS-derived T_h17_ cells was strongly correlated with *Ifng* and *Il2ra*, corresponding to our results from *in vitro* generated human cells, whereas *Foxp3, Il17a*, and *Il17f* were not correlated with *Csf2* (Figure S3A in Supplementary Material). LN-derived T_h17_ cells exhibited a different pattern, with *Csf2* correlating with *Il17a, Il17f*, and *Il2ra* but not *Ifng* (Figure S3B in Supplementary Material).

### GM-CSF^+^ CD4^+^ T Cells Comprise Several Subpopulations

The above data show that despite the presence of “GM-CSF only” single-positive cells (with respect to the markers studied herein), GM-CSF^+^ CD4^+^ T cells co-express a range of different other “subset-specific” CD4^+^ T cell markers. To gain insight to the heterogeneity and possible subpopulation structure of GM-CSF^+^ CD4^+^ T cells, we performed an unbiased computational analysis using t-SNE (23), a non-linear dimensionality reduction technique, followed by model-based clustering of single cells. First, we selected GM-CSF^+^ cells separately from both induction conditions (with or without TGF-β). Next, the multi-dimensional data containing intensity values of all other measured markers (except GM-CSF) was projected to two dimensions using t-SNE, and in the two-dimensional space cells were clustered using model-based clustering. In detail, we pre-gated on GM-CSF^+^ cells and studied their subpopulations based on expression levels of five other markers (FOXP3, CD25, IFN-γ, cell proliferation dye, IL-2). We determined that GM-CSF^+^ cells clearly comprised several subpopulations with different combinations of expression of the other markers (Figure 6D; Figure S4 in Supplementary Material). Furthermore, the subpopulations within GM-CSF^+^ CD4^+^ T cells induced by activation only (No cytokine) were clearly distinct with bimodal expression of the measured markers. However, subpopulations within GM-CSF^+^ CD4^+^ T cells induced by activation plus TGF-β appeared more connected, with a corresponding graded expression of FOXP3 (Figure 6D; Figure S4 in Supplementary Material). Taken together, the above data show that GM-CSF^+^ CD4^+^ T cells comprise several subpopulations co-expressing other T cell subset markers on the single-cell level. Of these, IFN-γ was most strongly correlated with GM-CSF independent of the condition, and TGF-β increased the fraction of GM-CSF^+^ cells co-expressing FOXP3.

### Cell Population Composition Changes throughout the Time Course of Differentiation

As described above, FOXP3 as well as IFN-γ harbored the strongest correlation with GM-CSF expression. To extract information about plausible trajectories of the differentiation of GM-CSF^+^ CD4^+^ T cells, we used labeling with a proliferation dye to track the number of cumulative cycles in each cell and used it as an approximation of the differentiation phase based on the assumption that more activated and differentiated cells have gone through more rounds of cell cycle. In each generation, we investigated the composition of the population with respect to the fractions in each quadrant defined by the FOXP3/GM-CSF and IFN-γ/GM-CSF pairs. We carried out this analysis separately on samples that were activated without any cytokine and samples that were activated in the presence of TGF-β1, since we observed that although the GM-CSF^+^ fraction was increased by TGF-β, in both conditions there were GM-CSF^+^ cells and they might represent different types of GM-CSF^+^ cells.

**Figure 7:**
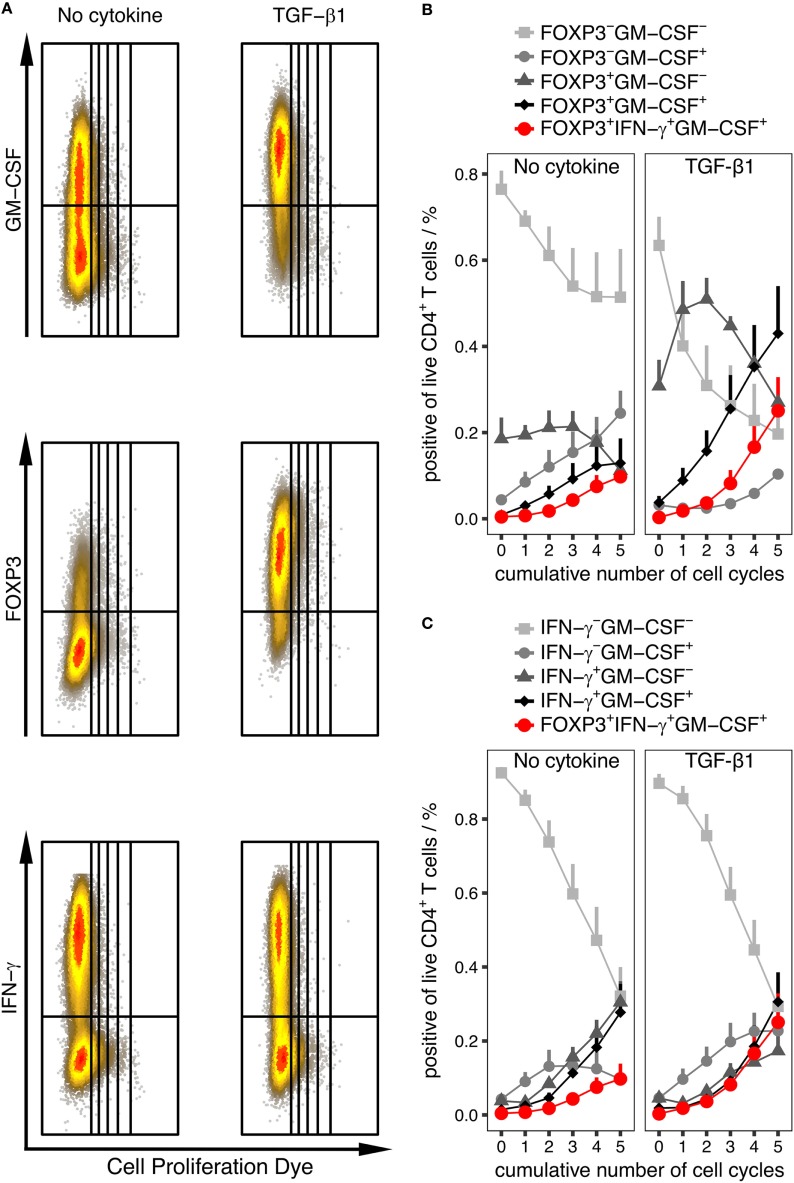
**Quantification of cell population subsets throughout differentiation phases represented by cumulative number of cell cycles**. Human naïve CD4^+^ T cells were labeled using Cell Proliferation Dye eFluor450 and were activated with anti-CD3/CD28 beads alone (No cytokine) or in the presence of “TGF-β1” for 6 days. The percentage of given subpopulations within live CD4^+^ T cells and within each cycle was measured by intracellular flow cytometry after restimulation with PMA/Iono/BFA. **(A)** Representative dot plots for each stimulation condition. **(B,C)** Percentage of cells in all quadrants using the GM-CSF and FOXP3 marker pair **(B)** or GM-CSF and IFN-γ marker pair **(C)** within each cycle are given. In addition, triple-positive cells for GM-CSF, IFN-γ, and FOXP3 are indicated in red in **(B,C)**. Data are presented as mean + SEM of six donors.

We determined that in both conditions only the double-negative fractions of IFN-γ^−^GM-CSF^−^ and FOXP3^−^GM-CSF^−^ decreased monotonously over generations, showing that the averaged expression of these markers increases as cells proliferate in both conditions (Figure 7). Interestingly, in the presence of TGF-β1, the FOXP3^+^GM-CSF^+^ fraction monotonously increased over generations whereas the FOXP3^+^GM-CSF^−^ fraction showed a unique distribution curve over the generations with an increase up to cycle 2 and decrease from cycle 2 to 5 (Figure 7B). Without TGF-β1 instead, the fraction of FOXP3^+^ cells was generally low but still, the shape of the population curves over generations was similar as without TGF-β1 (Figure 7B). The fractions positive for any combination of IFN-γ and GM-CSF generally increased over generations (Figure 7C). Taken together, these analyses suggest that expression of GM-CSF and IFN-γ occurs with similar kinetics, while FOXP3^+^ cells appear earlier than GM-CSF^+^ cells. Notably, also cells triple-positive for GM-CSF, IFN-γ, and FOXP3 were observed (Figures 7B,C), the fraction of which was low in the early cycles and increased in later stages, reaching a substantial fraction upon addition of TGF-β.

## Discussion

The main finding of this study is that TGF-β can enhance GM-CSF induction during human CD4^+^ T cell differentiation, and these GM-CSF^+^ CD4^+^ T cells comprise several subpopulations that co-express other T cell subset markers such as IFN-γ or FOXP3. Besides TGF-β, we have screened a range of cytokines and their combinations for their ability to induce GM-CSF. To our knowledge, the present study is the largest comparative study concerning GM-CSF-inducing conditions in human CD4^+^ T cells and the first example of employing a data-driven, unbiased analysis of GM-CSF-inducing conditions, GM-CSF^+^ cell subpopulation composition as well as correlation of GM-CSF with other markers. This study advances our understanding of the complexity of human GM-CSF^+^ CD4^+^ T cell induction and phenotype.

Illustrating the complexity of the differentiation of these cells, we also discovered that the effect of TGF-β on human GM-CSF^+^ CD4^+^ T cell differentiation was dependent on the type of TCR activation and co-stimulation. Specifically, with anti-CD3/CD28 bead-mediated T cell activation TGF-β enhanced GM-CSF^+^ CD4^+^ T cell differentiation, whereas upon pb anti-CD3 and soluble anti-CD28-mediated T cell activation TGF-β repressed GM-CSF. In our experiments, activation by anti-CD3/CD28 beads induced more T cell proliferation than the activation by pb anti-CD3 and soluble anti-CD28 antibodies. Nevertheless, the activation-dependent differential effect of TGF-β on human GM-CSF^+^ CD4^+^ T cell differentiation was not due to different extent of proliferation, because the TGF-β effect was observed in each cycle when conditioning on the number of cumulative cell cycles. The differential effect of TGF-β on GM-CSF induction between the two activation types may be due to differences in the strength of activation, difference in the ratio of anti-CD3 and anti-CD28 antibodies and/or differences in the basal GM-CSF expression without addition of cytokines. Our findings identify a novel, context-dependent role of TGF-β in the regulation of human CD4^+^ T cell differentiation.

Previous studies investigating the effect of TGF-β on human GM-CSF^+^ CD4^+^ T cell differentiation have reported either neutral (5) or inhibitory (9) effects, and those differences may be related to the experimental settings such as mode of T cell activation which, based on our data, may change GM-CSF induction. In both of these studies, the authors used 10 ng/ml TGF-β, pb anti-CD3-, and soluble anti-CD28-mediated T cell activation at similar concentrations and slightly different markers for defining and sorting human naïve CD4^+^ T cells. The cell culture medium may also influence T cell differentiation. While we used serum-free X-VIVO 15 medium, Zielinski and colleagues (5) used RPMI 1640 medium supplemented with 5% human serum and they observed that TGF-β had no effect on human GM-CSF^+^ CD4^+^ T cell differentiation (5). Because serum contains high levels of TGF-β that may be biologically active (37, 38), and bovine serum-derived TGF-β1 is 100% conserved compared to the human TGF-β1 protein, this might have precluded the possibility for the authors to study potential effects of exogenously added TGF-β (5). In the abovementioned study that described inhibitory effects of TGF-β on human GM-CSF^+^ CD4^+^ T cell induction, the authors did not specify which medium and whether serum was used (9). Importantly, our results also show that the GM-CSF induction upon TGF-β was influenced by the sodium concentration in the medium, which is affected by addition of serum and differs between X-VIVO 15 medium and RPMI 1640 medium (the latter containing 133 mM sodium). Further, IFN-γ^+^ cell fractions were generally higher with physiological compared to low sodium concentration within the range we tested. Because sodium has been suggested to drive “pathogenic” T_h17_ differentiation in EAE models, and pathogenic cells in EAE have been associated with GM-CSF and IFN-γ expression (6–8, 13, 18), we consider it important to study GM-CSF induction under “physiologic” sodium concentrations and to our knowledge, we are the first to study effects of sodium on GM-CSF induction without T_h17_-inducing cytokine conditions. To our knowledge, there is also no study that investigated the effect of TGF-β on GM-CSF^+^ T cell differentiation from human naïve CD4^+^ T cells in the presence of anti-CD3/CD28 beads, although T cell stimulation with such beads is similar to physiological activation because it mimics the interaction with antigen-presenting cells (39, 40).

It has further been suggested from studies in mice that TGF-β1 and TGF-β3 have differential effects, TGF-β3 inducing “pathogenic” GM-CSF^+^IL-17^+^ cells and TGF-β1 inducing GM-CSF^−^IL-17^+^ cells. Mechanistically, it was proposed that NaCl activates the kinase SGK1 that in turn increases IL-23R expression, consequently through IL-23R signaling enhancing the secretion of TGF-β3 which confers pathogenic properties to IL-17^+^ cells, including GM-CSF production (11, 13, 18). Contradicting this notion however, T cells from either Il23r^−/−^ or Sgk1^−/−^ mice cultured under T_h17_-polarizing conditions expressed increased *Csf2* levels compared to the wild-type counterpart (13), leaving the mechanism of sodium effects on GM-CSF unclear to date. Our data do not provide any evidence that TGF-β1 and TGF-β3 have differential qualitative effects on human GM-CSF expression, or any of the other markers we measured. This is in accordance with another study showing that TGF-β1 and TGF-β3 have similar effects on human GM-CSF^+^ CD4^+^ T cells (9), even though a different mode of T cell activation was applied here. Importantly, we did not observe an increased occurrence of GM-CSF^+^IL-17A^+^ cells in the presence of TGF-β3. Together, these data suggest that in contrast to the proposed mechanisms in the murine system (11), TGF-β3 does not seem to have a role in inducing specifically “pathogenic” human GM-CSF-producing (T_h17_) cells and did not affect human cells differentially as compared to TGF-β1. Another study performed in mice found that – also in the presence of IL-6 but with antigen-specific stimulation in contrast to *Ref* (11) – both TGF-β1 and TGF-β3 were unable to induce “pathogenic” murine T_h17_ cells, whereas myelin-specific T_h1_ cells and pathogenic T_h17_ cells – differentiated in the absence of TGF-β – produced GM-CSF as measured on the bulk population level (17). It remains to be determined whether GM-CSF^+^IFN-γ^+^ double-positive cells are driving the pathogenesis of murine EAE, and whether there are differences in the murine *versus* human system, although the latter is difficult to assess due to the impossibility of functional “pathogenicity” assays in humans.

IL-2 has been reported to enhance the differentiation of human GM-CSF^+^ CD4^+^ T cells independently of proliferation since the increased percentage of GM-CSF^+^ cells as a function of IL-2 concentration was present in every proliferation generation (5, 9). Since IL-2 is abundantly produced by T cells and also required for T cell activation and proliferation, it is difficult to study how the absence of IL-2 affects differentiation. In our experiments, the absence of exogenous IL-2 or blocking of IL-2 by neutralizing antibodies drastically reduced T cell proliferation when human naïve CD4^+^ T cells were activated by pb anti-CD3 and soluble anti-CD28 antibodies, but not when they were activated by anti-CD3/CD28 beads. The latter generally led to stronger activation, as assessed by CD25 upregulation and proliferation. This suggests that with bead stimulation the amount of endogenously produced IL-2 can overcome the lack of exogenous IL-2 supplementation and renders IL-2 neutralization insufficient. This possibility is consistent with our observation that addition of IL-2 in the presence of anti-CD3/CD28 bead-mediated activation affected neither the expression of GM-CSF nor the other markers we measured including proliferation. When we blocked IL-2 signaling by a pharmacological inhibitor of STAT5, proliferation was decreased indicating that IL-2 signaling was blocked, nevertheless GM-CSF^+^ fractions within in each proliferation cycle were unchanged suggesting that TGF-β enhances GM-CSF expression in cells activated by anti-CD3/CD28 beads independently of IL-2 signaling. Although GM-CSF^+^ production was generally higher in cells that underwent more proliferation cycles, the effect of different cytokines on GM-CSF expression was yet independent of proliferation, consistent with the results of previous studies on human GM-CSF^+^ cells (5, 9).

IL-7 and IL-21 belong to the same cytokine family as IL-2, and IL-7 has been suggested from mouse and human studies as an inducing cytokine of GM-CSF^+^ CD4^+^ T cells (9, 41). In the same way as IL-2, neither IL-7 nor IL-21 enhanced GM-CSF induction in our experiments with bead-bound anti-CD3/CD28 stimulation. This indicates that signaling *via* the common gamma chain cytokine receptor – which involves STAT5 – might generally affect GM-CSF expression differentially in an activation-type dependent manner.

IL-12 – that is known as a T_h1_-inducing cytokine – has been suggested from studies both in murine and human cells to induce GM-CSF^+^ CD4^+^ T cells (5, 17, 42). In our experiments with bead stimulation, IL-12 had no effect on GM-CSF induction but it increased IFN-γ expression as expected (data not shown), again highlighting the sensitivity of GM-CSF induction to differences in experimental conditions. Other IL-12 family members are IL-23 (that augments T_h17_ responses as described above) and IL-27 [that drives anti-inflammatory IL-10 responses (43)]. IL-27 has been demonstrated to inhibit GM-CSF production by both murine and human cells (5, 44). In our experiments, the anti-inflammatory cytokine IL-10, belonging to a different family of cytokines, did not have notable effects on GM-CSF expression.

T_h17_-driving cytokines such as IL-1β and IL-23 enhanced GM-CSF^+^ cell fractions in murine cells, and it was suggested that GM-CSF is co-produced with IL-17 (14). In our experiments (in the presence of bead activation), IL-1β had a weak inducing effect but IL-23 had a strong inhibitory effect on GM-CSF expression. This is in accordance with other studies on human cells using pb TCR activation (5, 9), suggesting that this effect – as opposed to the dual role of TGF-β in GM-CSF induction – is independent of the T cell activation mode, but might instead reflect differences between human and murine cells. Alternatively, the differences between mouse and human cells may be due to the tissue source: human T cells for *in vitro* culture studies are typically isolated from blood whereas murine T cells are commonly isolated from the spleen. Furthermore, it has to be considered that the *in vitro* activation and cytokine treatment of cells might not reflect the *in vivo* priming situation and in addition, cells might be changed by the environment at the inflammatory site, such as in the brain in the case of MS. Furthermore, we found that IL-6 (another T_h17_-inducing cytokine) had a strong inhibitory effect on GM-CSF expression, which is in agreement with a study inducing GM-CSF^+^ cells using pb CD3/CD28 activation (5) while in another study using such activation IL-6 had no effect (9). Overall, from our and other studies it appears that the differentiation requirements of human IL-17^+^ and GM-CSF^+^ CD4^+^ T cells are different and the only “T_h17_-inducing” factor that did not exert opposing effects for the two subsets was IL-1β, which interestingly is required for IL-17 induction specifically in human T cells (45–48). For therapeutic approaches and interpretation of murine disease models, particularly for MS, it will be highly relevant to further understand the differences in the differentiation requirements of murine and human IL-17^+^ and GM-CSF^+^ CD4^+^ T cells in the future.

To disentangle the relationship of GM-CSF with other major CD4^+^ T cell lineage markers, we systematically investigated the relationship between these in terms of their induction and repression requirements and their single-cell co-expression under several different cytokine conditions. From correlation and LASSO regularized regression analyses on the population level, it was apparent that GM-CSF had similar induction and repression requirements as FOXP3. However, when we investigated the correlation of GM-CSF and other markers in terms of their expression on the single-cell level, it was apparent that GM-CSF and IFN-γ were more co-expressed by the same cells than any other GM-CSF/marker pair that we studied. The co-expression relationship between GM-CSF and IFN-γ in our data was very robust across different cytokine stimulation conditions suggesting that possibly GM-CSF and IFN-γ have a shared intracellular regulation, possibly common transcription factors regulating them. Furthermore, the results suggest that GM-CSF and FOXP3 have similar inducing and inhibiting factors on the population level, but they are regulated rather independently within a single cell. While we observed a correlation of GM-CSF and FOXP3 on the population as well as single-cell level in the human system, such correlation was not found in our analysis of murine single-cell T_h17_ data (36), which might reflect species differences concerning activation-induced FOXP3 expression in human conventional T cells or be a result of the inhibitory effects of RORγt on FOXP3 in T_h17_ cells. Regarding IFN-γ and GM-CSF, our analysis of this independent murine single-cell RNA-sequencing dataset (36) suggests that the co-expression of IFN-γ and GM-CSF in the same cell is a feature that is true across species, and it might specifically signify potentially pathogenic cells derived from the CNS. At least considering the subset of IL-17A^+^ cells, our analysis further suggests that co-expression patterns of GM-CSF with other markers differ depending on the tissue. Notably, most IL-17A^+^ murine T cells did not co-express *Il17a* and *Csf2*, whether derived from LN or CNS (36). However, in human brain lesions from MS patients substantial fractions of GM-CSF^+^IL-17A^+^ and GM-CSF^+^IFN-γ^+^ T cells were detected using immunohistochemistry (49). Interestingly, a recent study in which Hafler and colleagues cloned single myelin-reactive T cells from MS patients and healthy donors also determined that many IFN-γ^+^ clones also produced GM-CSF, and most IL-17-producing clones from MS patients co-expressed GM-CSF – yet many T cell clones derived from MS patients that produced high levels of GM-CSF expressed neither IFN-γ nor IL-17 (10).

To characterize the possibly different subtypes of GM-CSF^+^ cells, we analyzed subpopulations of GM-CSF^+^ cells that were induced by activation only or by activation plus TGF-β applying t-SNE followed by model-based clustering. t-SNE revealed that GM-CSF^+^ cells induced by activation only had rather distinct groups as compared to GM-CSF^+^ cells induced with TGF-β. The different groups in the “No cytokine” condition had distinct levels of FOXP3, CD25, and IFN-γ, whereas the different groups in the “TGF-β” condition had graded levels of FOXP3 and CD25 expression, but IFN-γ was still bimodally distributed across the clusters. This suggests that in the presence of TGF-β, when FOXP3 is strongly induced, GM-CSF^+^ cells with slightly different FOXP3 levels are distinct based on other markers, whereas without TGF-β, FOXP3^+^GM-CSF^+^ cells are generally less frequent and form a distinct category. Interestingly, only when TGF-β was present, a rare population of FOXP3^high^CD25^high^ cells that did not produce IL-2 was observed, which might represent a population of Treg-like cells producing GM-CSF. Although these *in vitro* generated cells may be distinct from the situation *in vivo*, it is notable that murine *in vitro*-induced Tregs were demonstrated to produce IFN-γ and GM-CSF upon restimulation. However, these myelin auto-antigen-specific Tregs did not induce EAE but instead suppressed pathology *in vivo*, accompanied by loss of GM-CSF expression under inflammatory conditions (50). Interestingly, IL-6, IL-27, or a combination of IL-2 with TGF-β mediated this loss of GM-CSF expression in murine Tregs. Based on these results, it is tempting to speculate that GM-CSF^+^FOXP3^+^ cells might be a subset of Treg-like cells, or alternatively they might reflect activation-induced FOXP3 expression in human cells (51, 52).

We and others have extensively studied the immune-suppressive function of human *in vitro* TGF-β-induced FOXP3^+^ Treg cells (iTregs) (53), with conflicting results possibly due to differences in experimental setup during Treg induction or setup and controls used in suppression assays (34, 54, 55). Activation-induced low and transient FOXP3 expression in human activated conventional T cells further complicates such studies, which however mostly concur in the conclusion that (low) FOXP3 expression is insufficient to confer suppressive function. In a setup similar to the studies in the present work, we determined that despite high FOXP3 expression, iTreg suppressive function *in vitro* did not exceed unspecific “suppression” of FOXP3-negative control stimulated cells, except when iTregs were induced in the presence of TGF-β + retinoic acid + rapamycin (34). Nevertheless these TGF-β + retinoic acid + rapamycin iTregs did not suppress in a, though artificial, xenogeneic *in vivo* suppression assay (34), highlighting the complexity of assays for determining iTreg suppressive function. A major complication with assays using human iTregs is that these cells lack epigenetic demethylation in the so-called Treg-specific demethylated region (TSDR) in the FOXP3 locus (56, 57) and hence lose FOXP3 upon restimulation (34, 58). Therefore, it would be complicated to study whether GM-CSF^+^FOXP3^+^ cells would lose GM-CSF upon restimulation in analogy to the murine system in Ref. (50). It remains subject to future investigations to test whether the rare population of GM-CSF^+^FOXP3^high^CD25^high^IL-2^neg^ cells that we observed under TGF-β addition represents a population of suppressive Treg-like cells producing GM-CSF, testing of which would require the definition of a unique surface marker (combination) of this population to enable its sorting for functional assays. Interestingly, while TGF-β-induced FOXP3 in our study did not seem to be affected by sodium addition, it was previously shown that suppressive activity of Tregs is reduced in the presence of added sodium (X-VIVO 15 medium + 5% human serum + 40 mM NaCl) and interestingly, these Tregs upregulated IFN-γ and GM-CSF on gene and secreted protein level (19). Interesting aspects to study in the future would be the subpopulation composition and cytokine requirements of GM-CSF^+^ cells induced from naïve T cells of patients with MS, as well as potential differences in functionality regarding Treg-like FOXP3^+^ subpopulations compared to healthy donors.

In order to explore possible differentiation trajectories of GM-CSF^+^ cells, we used proliferation tracking as an approximation of differentiation stage. Interestingly, FOXP3^+^GM-CSF^−^ cells preferentially occurred in the earlier cycles whereas in the later cycles, when these cells decayed, FOXP3^+^GM-CSF^+^ cells increased. This suggests that possibly the cells stimulated by TGF-β first express FOXP3 and then acquire the expression of GM-CSF. Interestingly, particularly in late stages and under addition of TGF-β, also cells expressing all the three markers GM-CSF, IFN-γ, and FOXP3 emerged, the functionality of which remains to be determined. Individual expression levels of these markers per cell likely affects the functional outcome and might add an additional layer of complexity. Unfortunately, a study (10) that described that a similar fraction of T cell clones derived from MS patients and healthy donors co-expressed IFN-γ and GM-CSF did not include FOXP3 as an additional marker, which would be an interesting subject of future investigation to identify the *in vivo* occurrence and potential relevance of such triple-positive cells.

Overall, our study identifies an activation- and sodium-dependent role for TGF-β in the induction of human GM-CSF^+^ CD4^+^ T cells and highlights potential species differences and context dependencies of GM-CSF induction. Our integrative characterization of the human GM-CSF^+^ CD4^+^ T cell population by computational and experimental methods (59, 60) adds to our understanding of the complexity of this cell population. These results have important implications for emerging considerations regarding outcomes of low- or high-salt (Western) diet in the context of autoimmune diseases such as MS (61), as well as for future therapies to block GM-CSF in MS patients, which is already in a phase I clinical trial (62).

## Author Contributions

SÉ designed the project, designed and performed all experiments, analyzed all data, and wrote the paper; AS helped in project design, performance, and design of experiments and contributed to writing the paper; VK contributed to the statistical approaches used in the analysis of the data and the statistical interpretation of the outcome of the same; JA helped in project design and supervised research; JT helped in project design and supervised research. All authors read and edited the manuscript and approved the final version of the manuscript.

## Conflict of Interest Statement

The authors declare that the research was conducted in the absence of any commercial or financial relationships that could be construed as a potential conflict of interest.
