# Supplementary material for: TGF-β Affects the Differentiation of Human GM-CSF+ CD4+ T Cells in an Activation- and Sodium-Dependent Manner
Source: Front Immunol. 2016 Dec 23;7:603. doi: 10.3389/fimmu.2016.00603 (PMC5179518; doi:10.3389/fimmu.2016.00603)
Supplement: Supplementary file 1 [file Image_1.pdf]

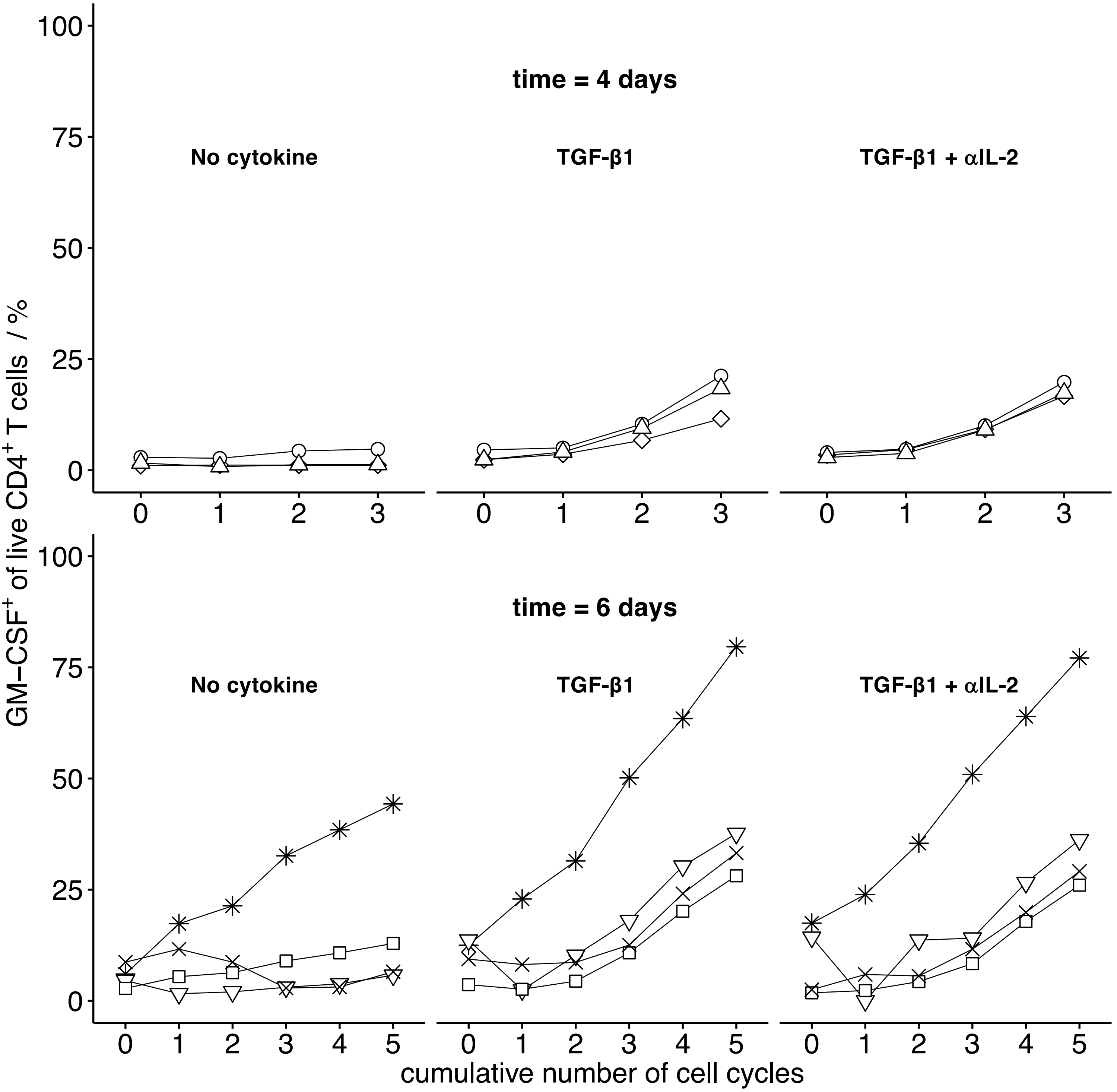

Supplementary Figure 1. Induction of human GM-CSF$^+$ CD4$^+$ T cells by TGF-β is independent of endogenous IL-2. Human naïve CD4$^+$ T cells were labeled using Cell Proliferation Dye eFluor450 and were activated with anti-CD3/CD28 beads alone ('No cytokine'), in the presence of TGF-β1 ('TGF-β1') or in the presence of TGF-β1 plus neutralizing anti-IL-2 antibody ('TGF-β1 + αIL-2') for the indicated time period. The percentage of GM-CSF$^+$ cells within live CD4$^+$ T cells within each cycle was measured by intracellular flow cytometry after restimulation with PMA/Iono/BFA. Percentage of GM-CSF$^+$ cells within the given proliferation cycles is shown for each stimulation and blocking condition. Each line and symbol represents a single donor.
