# Supplementary material for: TGF-β Affects the Differentiation of Human GM-CSF+ CD4+ T Cells in an Activation- and Sodium-Dependent Manner
Source: Front Immunol. 2016 Dec 23;7:603. doi: 10.3389/fimmu.2016.00603 (PMC5179518; doi:10.3389/fimmu.2016.00603)
Supplement: Supplementary file 2 [file Image_2.pdf]

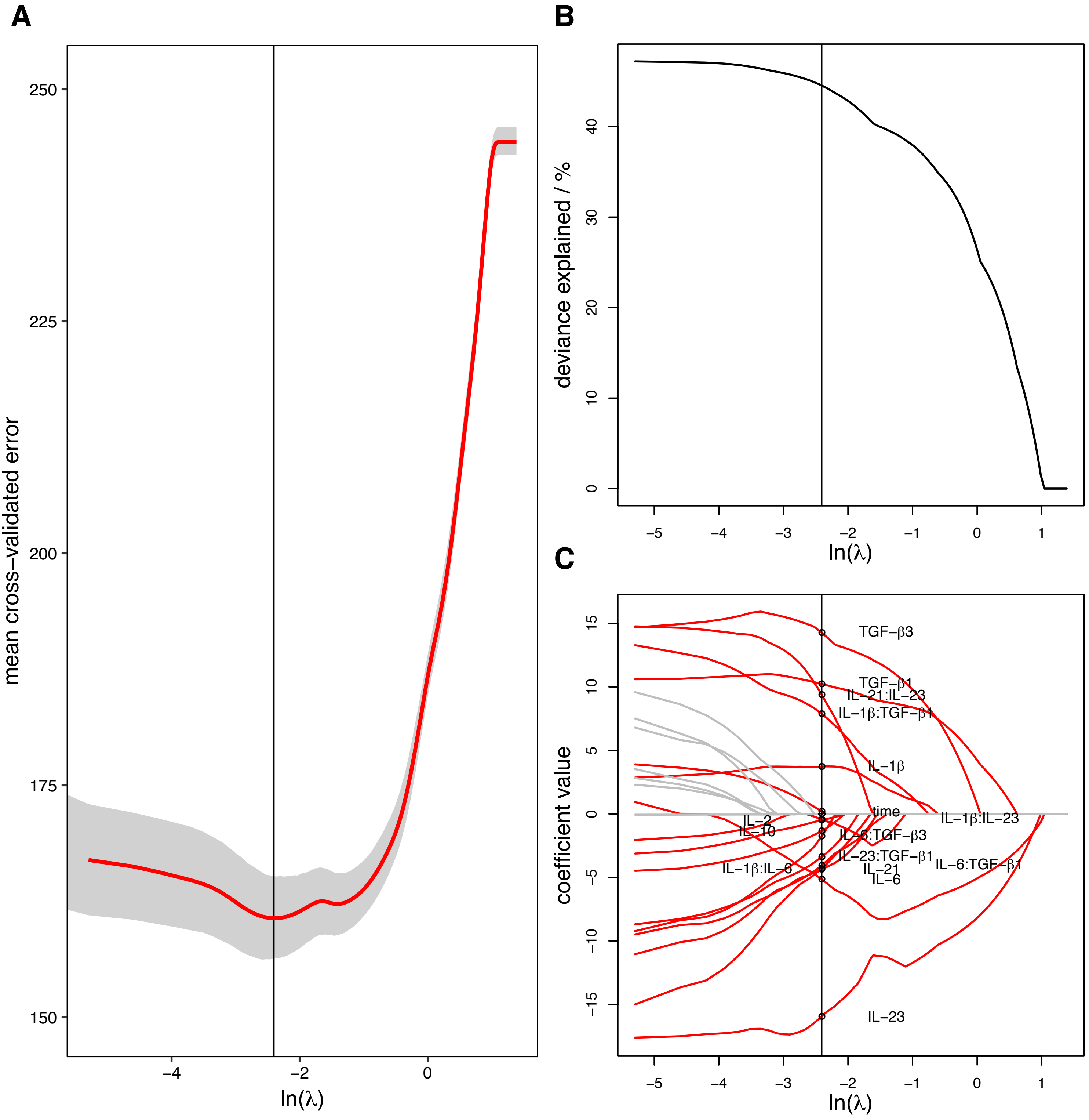

Supplementary Figure 2. Model selection using LASSO regularization. Linear regression was carried out where the change in the GM-CSF^{+} cell fraction within live cells compared to the control (activation without cytokines) was considered as target variable and the concentration of different cytokines and time were considered as predictors. In order to select a model and a set of predictors, LASSO regularization technique was applied. (**A**) 10-fold cross-validation was repeated 1000 times, each time with randomly chosen 10-fold splits; l denotes the penalty factor. Red line represents the mean of the mean cross-validated error (MCV) and the grey band shows the 0.1-0.9 quantiles of the MCV as the function of ln(l) from the 1000 repeated runs. Vertical line represents the ln(l) value where MCV has its local minimum (in the examined ln(l) range) i.e. the logarithm of l of the chosen model. (**B**) The percentage of deviance explained is shown as the function ln(l). Vertical line represents the logarithm of the penalty factor (l) of the chosen model. (**C**) The coefficient values are shown as the function of ln(l). Each line represents the coefficient of a given predictor and shows how it changes as a function of ln(l) throughout the model selection procedure. Vertical line represents the logarithm of the penalty factor (l) of the chosen model and circles represent the coefficient values of the predictors in this model. Predictors are indicated next to the circles.
