# Supplementary material for: TGF-β Affects the Differentiation of Human GM-CSF+ CD4+ T Cells in an Activation- and Sodium-Dependent Manner
Source: Front Immunol. 2016 Dec 23;7:603. doi: 10.3389/fimmu.2016.00603 (PMC5179518; doi:10.3389/fimmu.2016.00603)
Supplement: Supplementary file 3 [file Image_3.pdf]

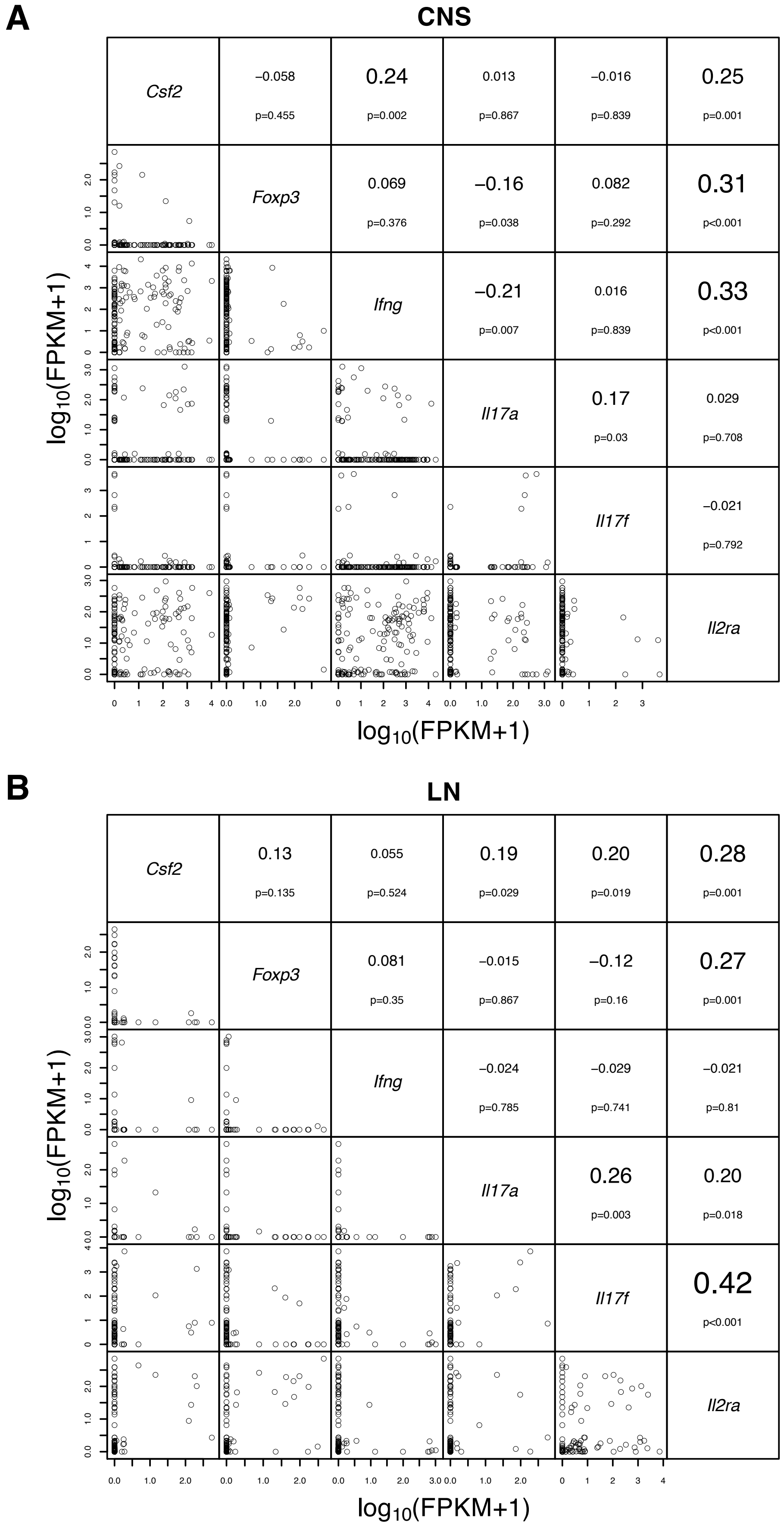


**Supplementary Figure 3. Correlation of *Csf2* with *Ifng*, *Foxp3*, *Il17a*, *Il17f* and *Il2ra* mRNA in single cells.** Single cell RNA-sequencing data from (Gaublomme et al., 2015) were analyzed and the Spearman correlation of *Cfs2* mRNA with mRNA of the indicated genes across single cells was calculated. Diagonal: Genes of the corresponding axis. Left to the diagonal: Each dot represents data from a single cell, and $\log_{10}$(FPKM+1) values are plotted as scatterplot. Right to the diagonal: Upper value denotes the Spearman correlation coefficient of the single cell mRNA expression levels for the given pairs of markers, lower value denotes the p-value calculated using a paired t-test considering the Spearman correlation coefficient (ρ) as random variable and testing the null-hypothesis that it is zero (H0: ρ=0). (**A**) Analyses performed on data from $T_{h17}$ cells sorted from the CNS of diseased mice with EAE, (**B**) Analyses performed on data from $T_{h17}$ cells sorted from the LNs of diseased mice with EAE.
