# Supplementary material for: TGF-β Affects the Differentiation of Human GM-CSF+ CD4+ T Cells in an Activation- and Sodium-Dependent Manner
Source: Front Immunol. 2016 Dec 23;7:603. doi: 10.3389/fimmu.2016.00603 (PMC5179518; doi:10.3389/fimmu.2016.00603)
Supplement: Supplementary file 4 [file Image_4.pdf]

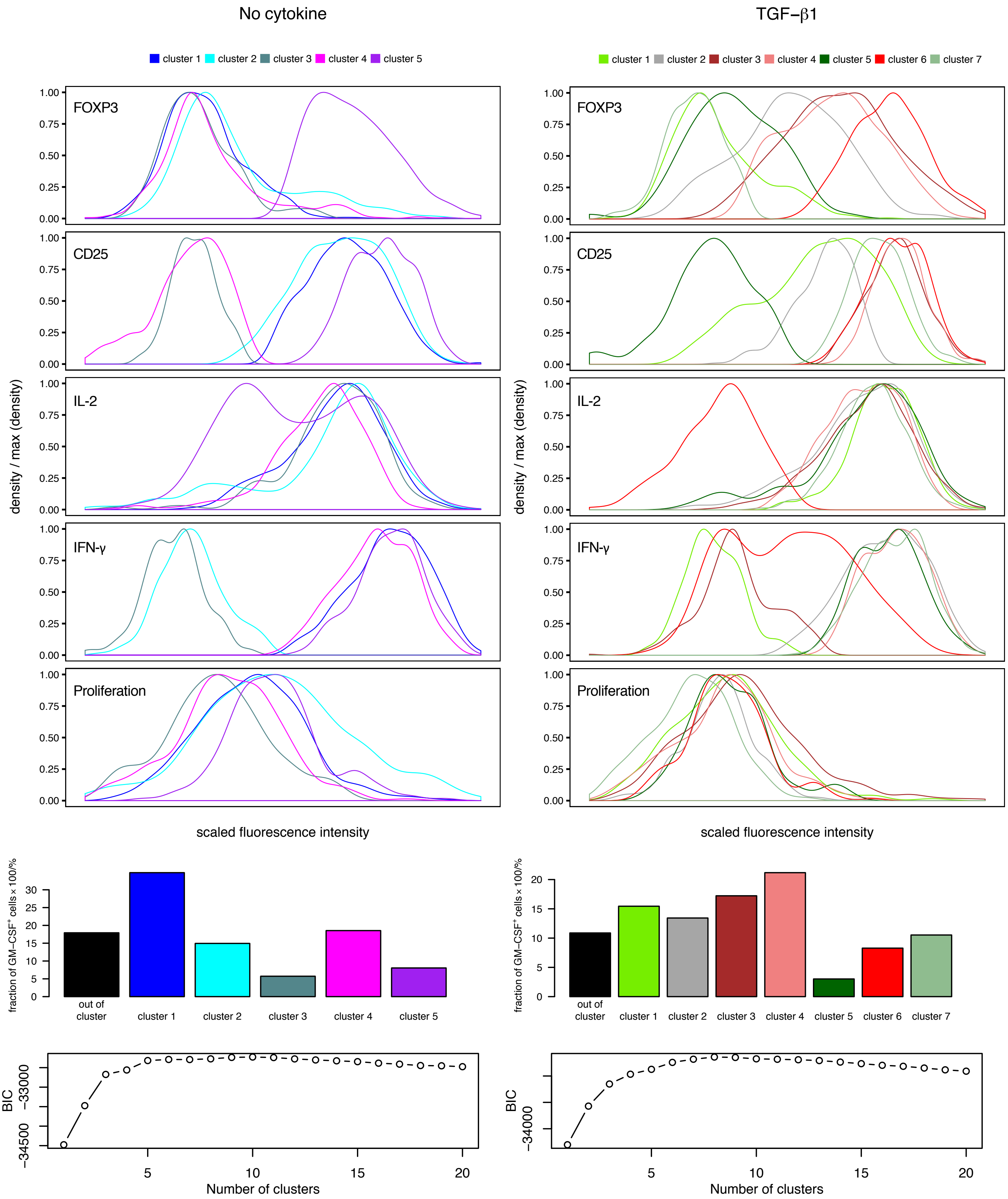

Supplementary Figure 4. Subpopulations based on t-SNE analysis and clustering. t-SNE analysis and model-based clustering of GM-CSF$^+$ cell subpopulations were performed as in Fig. 5D and the specific subpopulations indicated by the different colors are plotted as histograms separately for each of the markers (FOXP3, CD25, IL-2, IFN-γ and Proliferation Dye). The fraction of each subpopulation was also quantified and is given in the bar chart with the same color scheme. The dependency of the evaluation criterion for choosing the number of clusters (BIC: Bayesian Information Criterion) is shown as a function of the number of clusters. Cells were stimulated with anti-CD3/CD28 beads alone ('No cytokine', left panel) or with addition of TGF-β1 ('TGF-β1', right panel).
